# The role of spatial embedding in mouse brain networks constructed from diffusion tractography and tracer injections

**DOI:** 10.1016/j.neuroimage.2021.118576

**Published:** 2021-09-11

**Authors:** Scott Trinkle, Sean Foxley, Gregg Wildenberg, Narayanan Kasthuri, Patrick La Rivière

**Affiliations:** a Department of Radiology, University of Chicago, Chicago, IL, USA; b Department of Neurobiology, University of Chicago, Chicago, IL, USA

**Keywords:** Connectome, Diffusion MRI, Tractography, Neural tracer, Graph theory, Geometric networks

## Abstract

Diffusion MRI tractography is the only noninvasive method to measure the structural connectome in humans. However, recent validation studies have revealed limitations of modern tractography approaches, which lead to significant mistracking caused in part by local uncertainties in fiber orientations that accumulate to produce larger errors for longer streamlines. Characterizing the role of this length bias in tractography is complicated by the true underlying contribution of spatial embedding to brain topology. In this work, we compare graphs constructed with ex vivo tractography data in mice and neural tracer data from the Allen Mouse Brain Connectivity Atlas to random geometric surrogate graphs which preserve the low-order distance effects from each modality in order to quantify the role of geometry in various network properties. We find that geometry plays a substantially larger role in determining the topology of graphs produced by tractography than graphs produced by tracers. Tractography underestimates weights at long distances compared to neural tracers, which leads tractography to place network hubs close to the geometric center of the brain, as do corresponding tractography-derived random geometric surrogates, while tracer graphs place hubs further into peripheral areas of the cortex. We also explore the role of spatial embedding in modular structure, network efficiency and other topological measures in both modalities. Throughout, we compare the use of two different tractography streamline node assignment strategies and find that the overall differences between tractography approaches are small relative to the differences between tractography- and tracer-derived graphs. These analyses help quantify geometric biases inherent to tractography and promote the use of geometric benchmarking in future tractography validation efforts.

## 1. Introduction

The structural connectome (Sporns et al., 2005) is the comprehensive map of connections among all neurons in the brain. Constructing such a map represents a major frontier in neuroscience that relies on the development of novel imaging techniques across a range of spatial scales and model organisms and will provide insight into the basic function and development of the brain (Passingham, 2013) and its pathologies (Griffa et al., 2013). Diffusion MRI tractography is currently the only noninvasive method for mapping the human structural connectome (Yeh et al., 2020) and forms the basis of research initiatives such as the Human Connectome Project (Glasser et al., 2016). Together with mathematical tools from graph theory (Kaiser, 2011; Rubinov and Sporns, 2010; Sizemore et al., 2018; Yeh et al., 2020), tractography studies have helped reveal a number of important network properties in the human brain such as efficiency (Latora and Marchiori, 2001), modularity (Sporns and Betzel, 2016), and the organization of network hubs (van den Heuvel and Sporns, 2013).

Despite these advances, diffusion MRI also suffers from relatively poor spatial resolution and fundamental ambiguities in the mapping between the underlying white matter fiber orientations and the resulting diffusion signal (Schilling et al., 2017). Recent efforts to validate tractography have uncovered several limitations, and even modern approaches still produce high numbers of both false positive and false negative tracts (Aydogan et al., 2018; Maier-Hein et al., 2017; Schilling et al., 2019; Thomas et al., 2014). A specific limitation inherent to probabilistic streamline tractography is the fiber-length bias: local uncertainties in the fiber orientation distribution at each step in the tracking process accumulate to produce global errors for longer streamlines, leading to an underestimation of connectivity weights at greater distances. Characterizing this sort of geometric bias is complicated by the fact that the brain is itself a spatially embedded network with properties partially inherited from geometry: there is a metabolic wiring cost in the establishment of long-range connections, and true falloff of both structural and functional connectivity with distance has been shown with high-resolution invasive imaging as well as tractography in a number of mammalian species (Horvát et al., 2016; Markov et al., 2013; Perinelli et al., 2019; Roberts et al., 2016; Rubinov et al., 2015; Supekar et al., 2009). With tractography alone, it is challenging to distinguish the role spatial embedding plays in the true network properties of the brain from the potentially exaggerated representation of that geometric effect resulting from biases inherent to the imaging process.

To characterize methodological biases and improve tractography approaches, tractography experiments need to be validated with additional high-resolution imaging. Neural tracer data is the gold standard for mesoscale connectivity studies in a number of model organisms (Bota et al., 2015; Girard et al., 2020; Jbabdi et al., 2015; Markov et al., 2014; 2013; Oh et al., 2014). Data from the Allen Institute Mouse Brain Connectivity atlas (Oh et al., 2014) form the most comprehensive whole-brain mesoscale brain network in a mammalian species and have been used in previous tractography validation studies, primarily to characterize agreement in voxel-wise streamline density maps with consideration for the role of specific algorithm parameters (Aydogan et al., 2018; Calabrese et al., 2015; Chen et al., 2015). In this work, we expand the use of the Allen Institute tracer data as a validation tool for tractography connectomics in the mouse brain. Specifically, we use a graph-theoretical approach previously developed for human tractography data (Roberts et al., 2016) of benchmarking empirical brain graphs against an ensemble of random geometric surrogates. The random geometric surrogate graphs are constructed from each empirical graph in such a way that the node strength distribution and low-order relationships between connectivity and fiber distance are preserved, but the topology is otherwise destroyed, allowing for determination of the extent to which certain properties of the mouse structural brain network can be explained through spatial embedding alone.

Previous studies have also explored the influence of spatial embedding and geometry on the topology of the structural brain network in different mammalian species (Henderson and Robinson, 2013; Horvát et al., 2016; Markov et al., 2013; Roberts et al., 2016; Rubinov et al., 2015). While many of these studies explored the results in an evolutionary context in terms of pressures of wiring cost and efficiency (Bullmore and Sporns, 2012), our aim is instead to use the same tools across the two imaging modalities to specifically assess methodological geometric bias in tractography, taking the tracer-derived network as a significantly higher-fidelity representation of the true mesoscale connectome of the mouse brain. Furthermore, we explore the performance of different tractography approaches by assessing the use of two different streamline node assignment strategies. Accordingly, our analysis aims to do two things: (1) explore and compare the role spatial embedding plays in the topology of graphs derived from tractography and neural tracer imaging and (2) explore the extent to which graphs constructed from different tractography methods reflect the topological properties observed in the empirical tracer model.

## 2. Methods

### 2.1. Construction of the primary tracer graph

Our work uses a high-resolution model of the Allen Institute mouse brain structural brain network published by Knox et al. Knox et al. (2019) The Knox et al. model provides estimates of connectivity at the scale of 100 μm isotropic voxels in the Allen Mouse Brain Common Coordinate Framework (CCF v3), and is derived from 428 whole-brain anterograde fluorescent viral tracer experiments in wild-type C57BL/6J mice collected for the Allen Mouse Brain Connectivity Atlas (Oh et al., 2014). Underlying tracer data are available at http://connectivity.brain-map.org. The Knox et al. model can be accessed publicly through the repository available at https://github.com/AllenInstitute/mouse_connectivity_models. While derived from tracer experiments with injection locations that may span multiple distinct regions, the model allows for the efficient creation of regionalized connectivity models using custom brain parcellations based on labeled structures in the Common Coordinate Framework. For this study, we chose to define graph nodes as 286 of the 291 gray matter regions used to construct the regionalized voxel model analyzed in Knox et al. (2019). Five small regions were excluded due to being located almost exactly along the sagittal midline. A full list of gray matter structures used for the parcellation is available in Supporting Information Table S1. The model allows for four different connectivity metrics. Our tracer graph was constructed using the normalized connection density metric, which takes raw projection volume fractions and normalizes them by the volume of the source and target regions for each edge. The anterograde tracers used to produce the Knox et al. model provide a directed graph, whereas tractography graphs are derived from symmetric diffusion data and inherently produce undirected graphs. For fair comparison, the tracer graph was manually made to be undirected by summing all bidirectional connectivity between every pair of nodes. The Allen Mouse Brain Connectivity Atlas also assumes hemispheric symmetry, as all underlying tracer images were formed from injections into the right hemisphere. Hemispheric symmetry was manually enforced in the final graph in order to yield square connectivity matrices for subsequent analysis.

### 2.2. Additional tracer data

The Knox et al. model has been shown to outperform the homogeneous model originally published in Oh et al. (2014) and produces a graph that is nearly 100% fully connected at the scale of the parcellation used in this study. The true density of the whole mouse brain network is an ongoing subject of research. Initial modeling of the Allen Institute data estimated cortical density to vary from 32–52% (Oh et al., 2014) to 59–73% (Ypma and Bullmore, 2016). A recent study by Gămănuţ et al. (2018) observed a much higher cortical density in the mouse brain of 97% using tract-tracing measurements from independent retrograde tracer data. While only available for select ipsilateral intra-cortical edges, the Gămănuţ, et al. data have the benefit of representing empirical neuron counts that do not rely on the fixed parcellation template and downstream computational modeling required for the Knox et al. model. Accordingly, our cortical tractography connectivity weights were further benchmarked against these additional weighted connectivity data provided by the authors. These edge-weight values correspond to neuron count fractions within manually defined gray-matter region boundaries corresponding to the parcellation from the Allen Institute (Figure S6B in Gămănuţ et al. (2018)). As with the Knox et al. model, the Gămănuţ, et al. cortical subgraph was manually made to be undirected by summing bidirectional connectivity weights between every pair of nodes.

### 2.3. Animal procedures

Procedures for the collection of the diffusion MRI data used for this study have been published in a previous study (Foxley et al., 2020) and are repeated here for completeness. All procedures performed on animals followed protocols approved by the Institutional Animal Care and Use Committee and were in compliance with the Animal Welfare Act and the National Institutes of Health Guide for the Care and Use of Laboratory Animals. Five adult mice were deeply anesthetized with 60 mg/kg pentobarbital and sacrificed by intercardial perfusion with a solution (pH 7.4) of 0.1 M sodium cacodylate and heparin (15 units/ml). This was immediately followed by a solution of 2% paraformaldehyde, 2.5% glutaraldehyde, and 0.1 M sodium cacodylate (pH 7.4). Brains were carefully removed from the skulls and post-fixed in the same fixative overnight at 4 °C. Brains were soaked in phosphate buffered saline (PBS) prior to imaging for at least 72 h to remove fixative from the tissue.

### 2.4. Diffusion MRI acquisition

Resected mouse brains were dried of excess PBS and placed in 10 ml Falcon tubes. Tubes were filled with Fluorinert (FC-3283, 3M Electronics) for susceptibility matching and to improve shimming. Data were acquired at 9.4 T (20 cm internal diameter, horizontal bore, Bruker BioSpec Small Animal MR System, Bruker Biospin, Billerica, MA) using a 6 cm high performance gradient insert (maximum gradient strength: 1000 mT/m, Bruker Biospin) and a 35 mm internal diameter quadrature volume coil (Rapid MR International, Columbus, Ohio). Third-order shimming was iteratively performed over an ellipse that encompassed the entire brain, but did not extend beyond the boundaries of the Falcon tube/Fluorinert interface, using the Paravision mapshim protocol. Diffusion MRI was performed using a standard diffusion-weighted 3D spinecho sequence (TR = 600 ms, TE = 11.389 ms, b-value = 3000 s/mm^2^, *δ* = 3.09 ms, Δ = 6 ms, spatial resolution = 125 μm isotropic, number of b0s = 8, number of directions = 30, receiver bandwidth = 150 kHz, duration = 36h 28min 48s).

### 2.5. Diffusion MRI processing

Data and diffusion gradient vectors were manually reoriented to the standard RAS neurological display convention. Subsequent processing was performed with the MRtrix3 software package (Tournier et al., 2019). Data were denoised using the dwidenoise protocol (Veraart et al., 2016a; 2016b). Binary brain masks were generated for subsequent processing using the dwi2mask routine. The data were first fit to a diffusion tensor model (Basser et al., 1994) to calculate the fractional anisotropy metric (Basser and Pierpaoli, 1996). The data were then fit to fiber orientation distribution functions (fODFs) using constrained spherical deconvolution (Tournier et al., 2017; 2004) up to a maximum spherical harmonic order of *ℓ*_max_ = 6 (28 coefficients). The fractional anisotropy image from each dataset was spatially registered to the Allen reference mouse brain template at an isotropic voxel size of 100 μm using affine and diffeomorphic transformations calculated with the ANTS registration package (Avants et al., 2008; Klein et al., 2009). The Allen template and structure-level annotations in the Common Coordinate Framework were accessed using the allensdk software tool (https://allensdk.readthedocs.io). The spatial transforms calculated in ANTS were then applied to the fODFs using the mrtransform protocol in MRTrix3, which applies appropriate reorientation (Raffelt et al., 2012b) and modulation (Raffelt et al., 2012a) of the fODFs in order to preserve fiber densities across each lobe after transformation.

### 2.6. Construction of tractography graphs

Probabilistic tractography was performed in MRTrix3 using the iFOD2 algorithm (Tournier et al., 2010) (step size = 12.5 μm, maximum curvature = 30 μm, minimum length = 0.5 mm, maximum length = 30 mm, fODF cutoff = 0.055). Streamlines were seeded uniformly throughout each of the 286 gray matter regions in the right hemisphere used in the regionalized tracer model, with 2000 seeds per voxel, amounting to around 400 million total streamlines per dataset. Edge weights were determined from each tractography dataset using two different streamline node assignment strategies in order to compare their effects on downstream network structure. For “endpoint” connectivity, streamlines were assigned to the two nodes corresponding to the gray matter regions closest to their endpoints, within a maximum radius of 125 μm, corresponding to the size of the underlying diffusion data voxels. For “dense” connectivity, streamlines were assigned to all node-pairs corresponding to pairs of gray matter regions they traverse, not just those corresponding to their endpoints. Edge-weight values between two nodes were then taken to be the number of streamlines assigned to the two nodes under both endpoint and dense assignment strategies, resulting in two different graphs per tractography dataset. To match the normalized connection density metric used for the tracer graph, the weights for each node pair were then divided by the product of the two node volumes. As with the tracer graph, hemispheric symmetry was manually enforced to create square connectivity matrices. Also similar to the tracer graph, the probabilistic tractography seeding used in this work led to nearly fully connected graphs.

### 2.7. Construction of surrogate graphs

The goal for the construction of geometric surrogate graphs was to create an ensemble of graphs that preserve both the distribution of node strengths (the sum of weights at each node) and the low-order weight-distance relationships of a given empirical graph but are otherwise random. Geometric surrogate graphs were constructed from all empirical tracer and tractography graphs by directly following the methodology described in Roberts et al. (2016), repeated here for completeness. First, the fiber distance between each pair of nodes was quantified based on tractography results. The distance *f*_*ij*_ between nodes *i* and *j* was defined as the length of the shortest streamline connecting them, averaged across all datasets. To estimate first-order weight-distance effects, we follow Roberts et al. in fitting the logarithm of the edge-weights *w*_*ij*_ to a curve given by log *w*_*ij*_ ≈ *g*(*f*_*ij*_), where *g* is a cubic polynomial. This relationship was subtracted from the raw weights, and an additional parabolic curve was then fit to the residuals. After normalizing by this second curve, low-order distance-dependent effects were effectively removed from the weight values and they were randomly shuffled. After randomization, the transformations were applied in reverse order to reimpose low-order weight-distance effects. The original weight values were then reordered to match the random rank order of the surrogate weights. Finally, the node-strength distribution was restored using an iterative procedure that updates the sums of the rows and columns of the surrogate weight matrix towards the empirical values.

This procedure resulted in an ensemble of geometric surrogate graphs *W*_geo_ for each network construction method that preserve the low-order distance-dependent characteristics and node-strength distribution of the underlying empirical graph *W*_emp_, but have all other topological properties destroyed. We make the assumption that network properties that are preserved in the geometric surrogate graphs represent those that have been inherited from the spatial embedding of the brain. Likewise, we assume that differences in network properties between empirical and geometric surrogate graphs represent the extent to which those properties arise from other, non-geometric factors.

A similar procedure without the use of distance transformations was used to construct an ensemble of random surrogate graphs *W*_rand_, which preserve the exact strength-sequence of the underlying empirical graphs, but are otherwise completely random. The geometric surrogates *W*_geo_ represent the null hypothesis that topological properties of brain networks arise from the falloff of edge weights with distance for a given node-strength distribution, while the random surrogates *W*_rand_ represent the null hypothesis that topological properties of brain networks arise solely from the particular distribution of node strengths and locations.

## 3. Results

Here we report analysis of brain networks constructed using tracer imaging data and two tractography approaches: “endpoints” and “dense” corresponding to two methods of streamline node assignment. For each metric, our goal is to explore how graphs from each tractography approach compare to the tracer graph, specifically with respect to the relationship between empirical and geometric surrogate graphs. Unless otherwise noted, all results labeled “tracer” correspond to the whole-brain graph derived from the Knox et al. connectivity model. All analysis was performed in Python, with graph theoretical measures calculated using the networkx package (Hagberg et al., 2008).

### 3.1. Comparison of edge-weight values

**Fig. 1.**
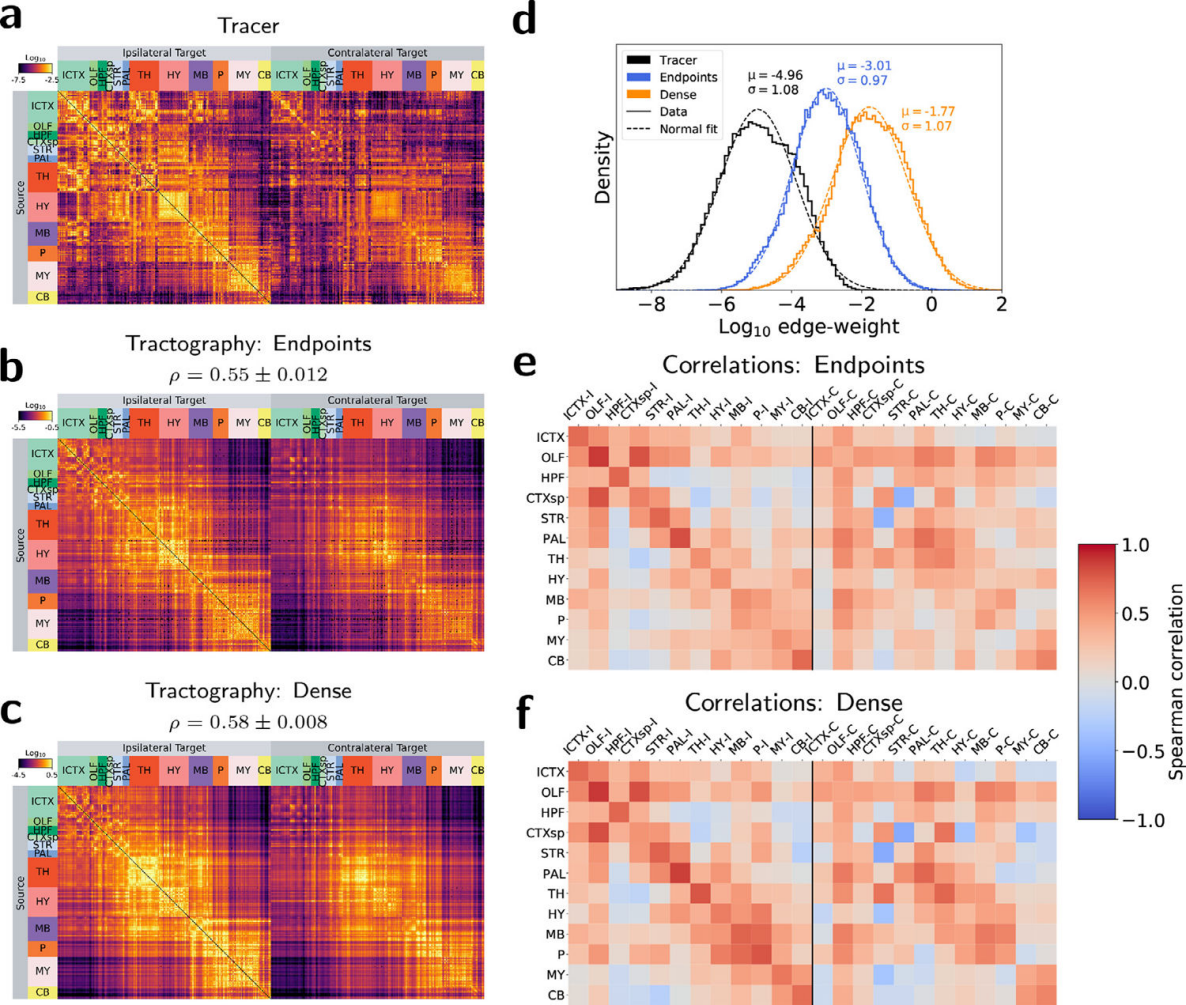
Edge-weight values. (a–c) Connectivity matrices for (a) tracer, (b) endpoint tractography, and (c) dense tractography. Rows represent nodes comprised of 286 gray matter regions across 12 major brain divisions. Columns represent the same nodes for ipsilateral (left) and contralateral (right) connections. Values are shown on a log-color scale spanning five orders of magnitude centered on the mean edge-weight value for each matrix. *ρ* values represent Spearman rank correlation coefficients between tractography and tracer weights across the whole brain. (d) Edge-weight distributions. Empirical weight histograms (solid lines) with corresponding normal fits (dashed lines). (e–f) Spearman correlations between tracer and (e) endpoint and (f) dense tractography edge-weights across 12 major brain divisions: Isocortex (ICTX), Olfactory areas (OLF), Hippocampal formation (HPF), Cortical subplate (CTXsp), Striatum (STR), Pallidum (PAL), Thalamus (TH), Hypothalamus (HY), Midbrain (MB), Pons (P), Medulla (MY), Cerebellum (CB). “-I” and “-C” in (e–f) refer to ipsilateral and contralateral correlations, respectively. All tractography values represent averages across 5 datasets.

Structural connectivity matrices and edge-weight distributions are shown for all empirical graphs in Fig. 1a–c. Differences in the mean weight values across tractography approaches in Fig. 1d follow predictable trends: dense node assignment resulted in higher weights than endpoint node assignment. Note that the physical interpretation of edge weights differs between modalities, so direct comparison of the edge-weight means between modalities is not meaningful. Tracer weights reflect normalized projection volumes and tractography weights reflect normalized streamline counts under different node assignment strategies. Regardless, edge-weight distributions had a comparable and approximately log-normal shape for all empirical graphs.

Overall agreement in edge-weight values was assessed using the Spearman rank correlation coefficient, a nonparametric correlation metric used in previous studies (Calabrese et al., 2015; Coletta et al., 2020) to assess nonlinear agreement between connectivity values. Spearman correlation values between tracer and tractography weights across all edges are shown above each tractography matrix in Fig. 1b–c. Edge weights constructed with dense node assignment had a slightly higher Spearman correlation with the tracer weights than those constructed with endpoint node assignment. The difference in correlations was statistically significant (*p* < 0. 01) using a *t*-test. Scatterplots of edge weights between the tracer and tractography graphs are available in Supporting Information Figure S1.

Spearman correlations between tractography and tracer weights assessed at the level of major brain divisions are shown in Fig. 1e–f. All tractography methods showed relatively high correlations in ipsilateral intra-division connectivity (diagonals in Fig. 1e–f), and weaker contralateral connectivity to homologous regions, reflecting not only a falloff in weight for longer-distance connections, but a falloff in agreement with tracer values. Dense node assignment led to higher Spearman correlations than endpoints for nearly all connections to the pons, and for connections between the hypothalamus, midbrain, and medulla.

### 3.2. Comparison of weight-distance relationships

The raw weight-distance distributions and polynomial fits used to construct the geometric surrogate graphs are available in Supporting Information Figure S2a–b. Transformed weights (Figure S2c) show effectively zero correlation with fiber distance using both Pearson and Spearman correlation coefficients, indicating that low-order distance relationships have been effectively removed prior to randomization for the construction of the geometric surrogate graphs.

**Fig. 2.**
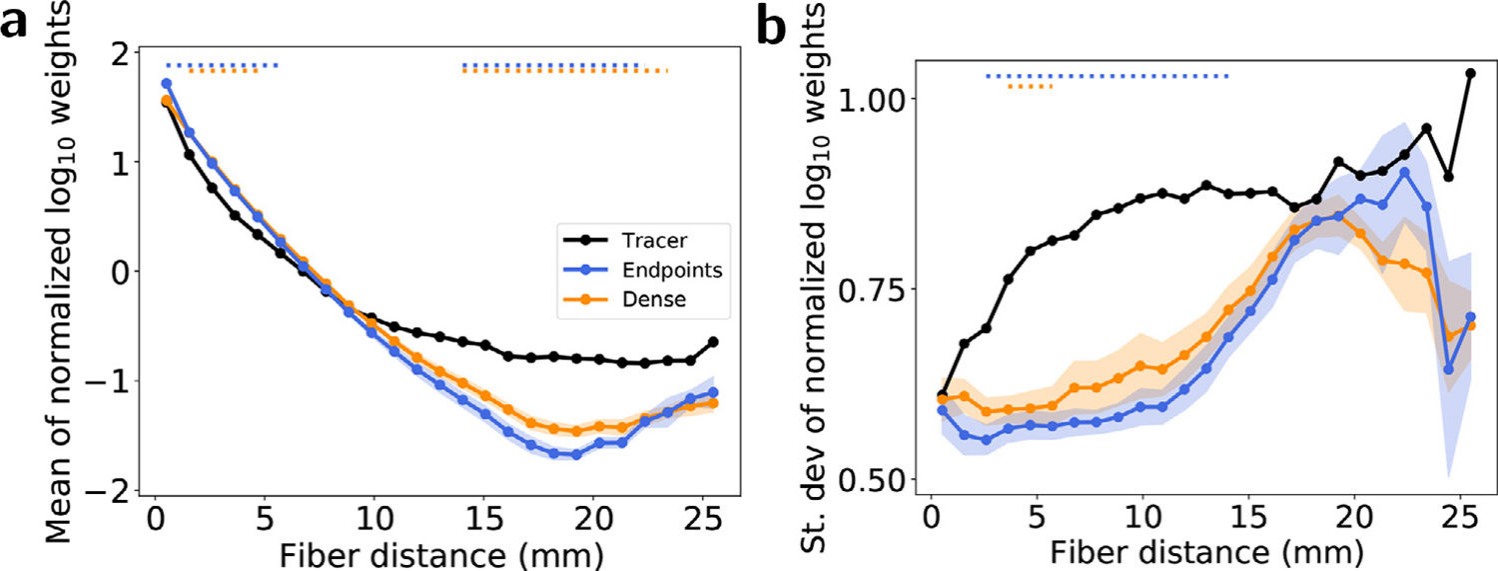
Normalized weight-distance relationships. (a) Circles represent the average log-weight z-scores for each method within 1 mm fiber distance bins. (b) Circles represent the standard deviation of the log-weight z-scores for each method within 1 mm fiber distance bins. Shaded regions represent 1 standard deviation across 5 tractography datasets. The widths of the horizontal lines at the top of each subfigure indicate the range of fiber distance bins with statistically significant differences (*p* < 0. 01) between tracer and tractography values for each tractography method, calculated using a one-sample *t*-test after correcting for multiple comparisons.

Because the weight values have different physical interpretations for each network construction method, the weight-distance relationships cannot be directly compared. Instead, Fig. 2 visualizes the relative relationships between methods after the log-weights were first standardized to zero mean and unit variance. Fig. 2a shows the relative falloff of mean normalized log-weights with fiber distance, and Fig. 2b shows the change in the standard deviation of normalized log-weights with fiber distance for each method.

The mean normalized weight-distance curves from both tractography graphs in Fig. 2a fall below the corresponding tracer curve for distances above around 10 mm, with both tractography methods showing statistically significant differences from tracers for fiber distance between around 17–22 mm. These results suggest that the tractography methods explored in this work underestimate relative long-range connectivity by as much as two orders of magnitude. For most fiber distances, both tractography methods also underestimate the standard deviation of normalized weights compared to tracers. A smaller relative standard deviation around the mean weight-distance relationship is consistent with tractography weights being more strictly determined by fiber distance than tracer weights are, though these differences were only statistically significant between around 3–15 mm for endpoint node assignment and for a smaller range around 4–6 mm for dense node assignment.

### 3.3. Comparison of network organization

#### 3.3.1. Modular structure

The modular structure of each brain graph was determined by optimizing the modularity (*Q*) using the Louvain algorithm (Blondel et al., 2008). Modularity expresses the extent to which a graph can be subdivided into distinct modules such that intra-modular connectivity is maximized and inter-modular connectivity is minimized (Newman, 2004). The confusion matrices for consensus node-module assignments are shown in Supporting Information Figure S3 for comparisons between empirical tractography and tracer modules as well as between empirical and geometric surrogate modules for all methods. Consensus node-module assignments represent the module ID label most frequently assigned to each node across 5 tractography datasets and ensembles of 100 geometric surrogates. ID labels were first standardized across tractography and geometric surrogate graphs by assigning labels to identified modules such that the overall agreement with the identified modules in the empirical tracer graph was maximized.

**Fig. 3.**
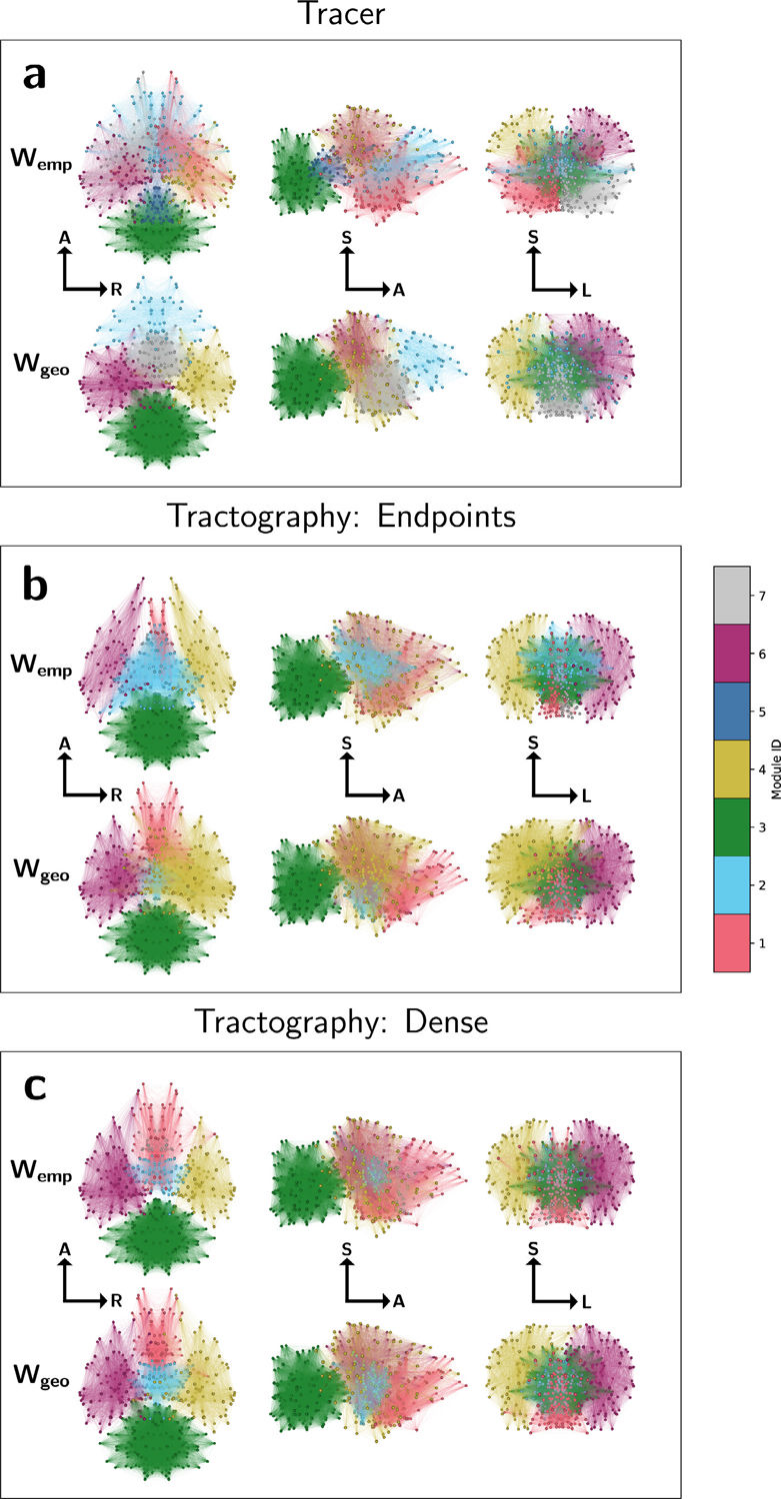
Module diagrams for (a) tracer, (b) endpoint, and (c) dense graphs in physical coordinates. Spheres represent the center of mass of each node, colored by module assignment. Intra-module edges are shown as colored lines. Graphs are visualized along the axial (left), sagittal (middle), and coronal (right) planes with labeled orientations: A = anterior, S = superior, R = right, L = left. Module assignments represent consensus values across 5 empirical tractography graphs and an ensemble of 100 geometric surrogate graphs.

The resulting consensus modular decompositions are visualized in physical coordinates for the empirical and geometric surrogate graphs derived with all network construction methods in Fig. 3. Spheres represent the physical location of distinct gray-matter nodes, which are colored according to their identified module. Intra-module edges are also visualized as colored lines. Overall, modules identified in the tractography networks are much more spatially clustered together than those in the tracer network. Intra-module edges are more likely to be shorter range for both tractography methods than for tracers, consistent with tractography modular structure being partially determined by geometric bias against long-range connections. This result is further quantified in Table 1, which shows the percent agreement in node-module assignment for pairs of graph construction methods. Graphs from both tractography methods show only modest agreement in module assignment with the tracer graph. The tracer graph also shows only modest agreement in module assignment with its geometric surrogates, suggesting that modules in the true mouse brain network are less spatially clustered than they would be if determined by geometry alone, while both tractography methods show much higher overlap in module assignment between their empirical and geometric surrogates.

**Fig. 4.**
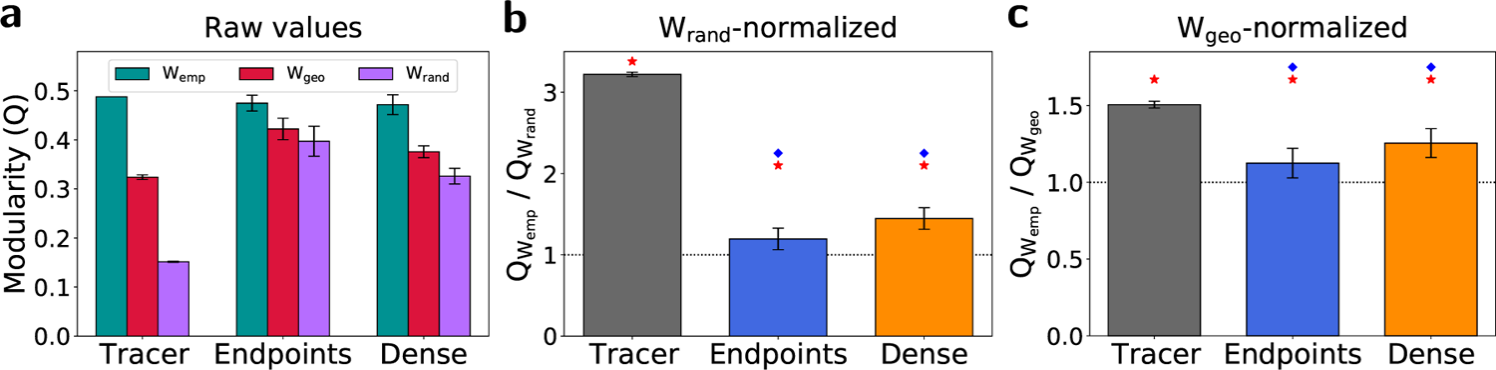
Modularity. (a) Raw *Q* values for empirical (*W*_emp_), geometric surrogate (*W*_geo_), and random (*W*_rand_) surrogate graphs. Error bars represent 1 standard deviation across 5 tractography datasets for empirical graphs, and 1 standard deviation across ensembles of 100 geometric and random surrogate graphs per method. (b) Empirical *Q* values normalized by their corresponding mean random surrogate *Q* value. (c) Empirical *Q* values normalized by their corresponding mean geometric surrogate *Q* value. Red stars indicate statistical significance (*p* < 0. 01) in the difference between *W*_emp_ and *W*_geo_ data calculated with Tukey’s range test, and blue diamonds indicate statistical significance (*p* < 0. 01) in the difference between *W*_rand_- and *W*_geo_-normalized tracer and tractography data, calculated with a permutation test.

The optimized *Q* value itself is a metric of network segregation, indicating a capacity for specialized processing to occur in different regions of the brain. Raw *Q* values are shown for all graphs in Fig. 4a. Figure 4b shows the empirical *Q* values normalized by the *Q* values from their corresponding random surrogate graphs, *W*_rand_, constructed by randomly shuffling weights within each empirical graph such that the node strength sequence is preserved. While raw *Q* values are comparable between the tracer and two tractography graphs, the tracer graph shows a substantially higher *W*_rand_-normalized modularity relative to all tractography methods, suggesting that tractography graphs under-represent the modularity of the mouse structural brain network beyond what would be expected from a random graph with the same strength sequence. In Fig. 4c, empirical *Q* values have been normalized by the *Q* values from their corresponding geometric surrogate graphs. This ratio represents the additional modular structure present in the empirical graphs beyond what would be predicted by spatial embedding alone, with a ratio of 1 indicating complete geometric determination. Both tractography methods show values significantly closer to 1 than the tracer graph does. Overall, these results suggest that modular structure in the mouse structural brain network is both underestimated overall and more geometrically determined in tractography relative to neural tracer imaging.

#### 3.3.2. Hub node organization

**Fig. 5.**
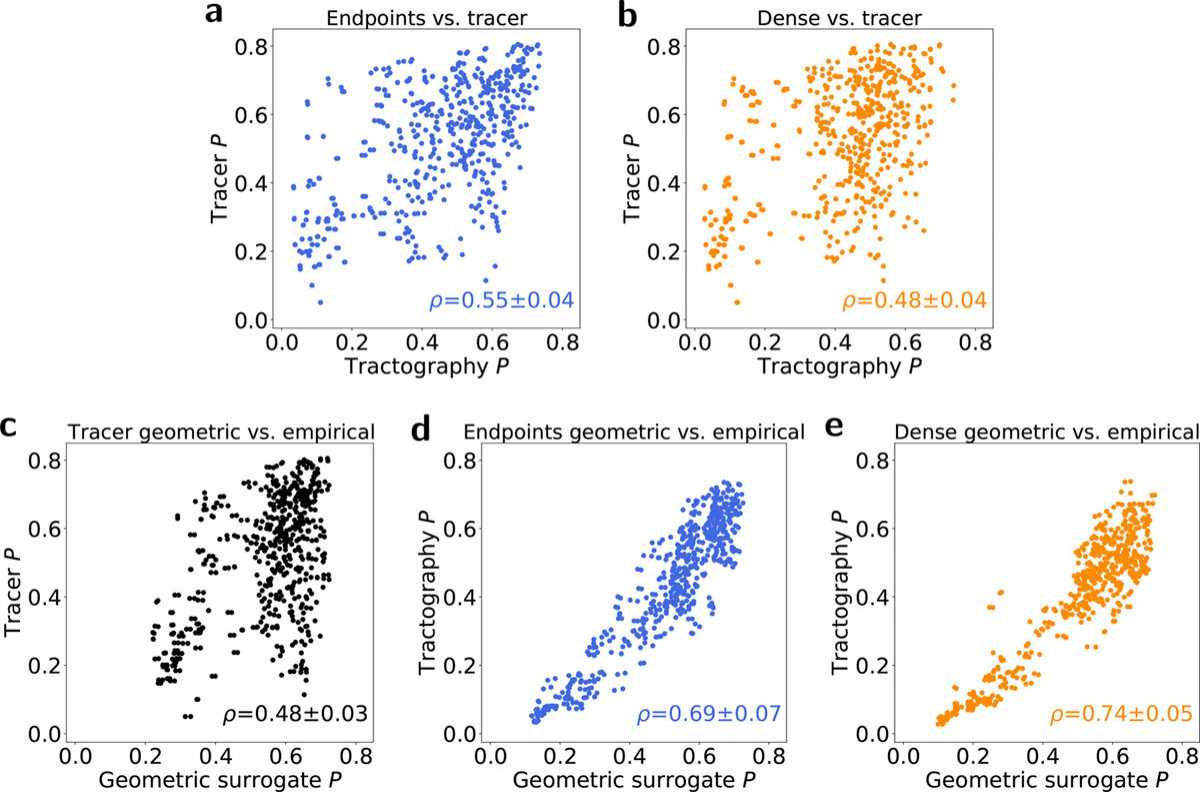
Scatterplots showing the relationship between participation coefficients assigned to each node by different network construction methods. (a–b) Correlations between empirical tracer and empirical (a) endpoints and (b) dense tractography participation coefficients. (c–e) Correlations between empirical (c) tracer, (d) endpoint, and (e) dense participation coefficients and those from their corresponding geometric surrogate graphs. Values represent averages across 5 tractography datasets and an ensemble of 100 geometric surrogate graphs.

The arrangement of the subnetwork of central “hub” nodes is key to understanding overall brain network structure. Hub nodes can be identified using a number of complementary centrality measures. The participation coefficient *P* is based on a particular modular decomposition and expresses the diversity of intermodular connections for a given node, with a value of 1 indicating a node is connected uniformly to all modules and a value of 0 indicating a node is connected exclusively to its own module (Guimerà and Amaral, 2005; Newman, 2004). Fig. 5 shows scatterplots of participation coefficients for empirical tractography and tracer graphs (Fig. 5a–b) and for each empirical graph method and its geometric surrogates (Fig. 5c–e). Both tractography methods show only weak correlation with the values from the corresponding tracer graph, but significantly stronger correlations with the values from their own geometric surrogates.

**Fig. 6.**
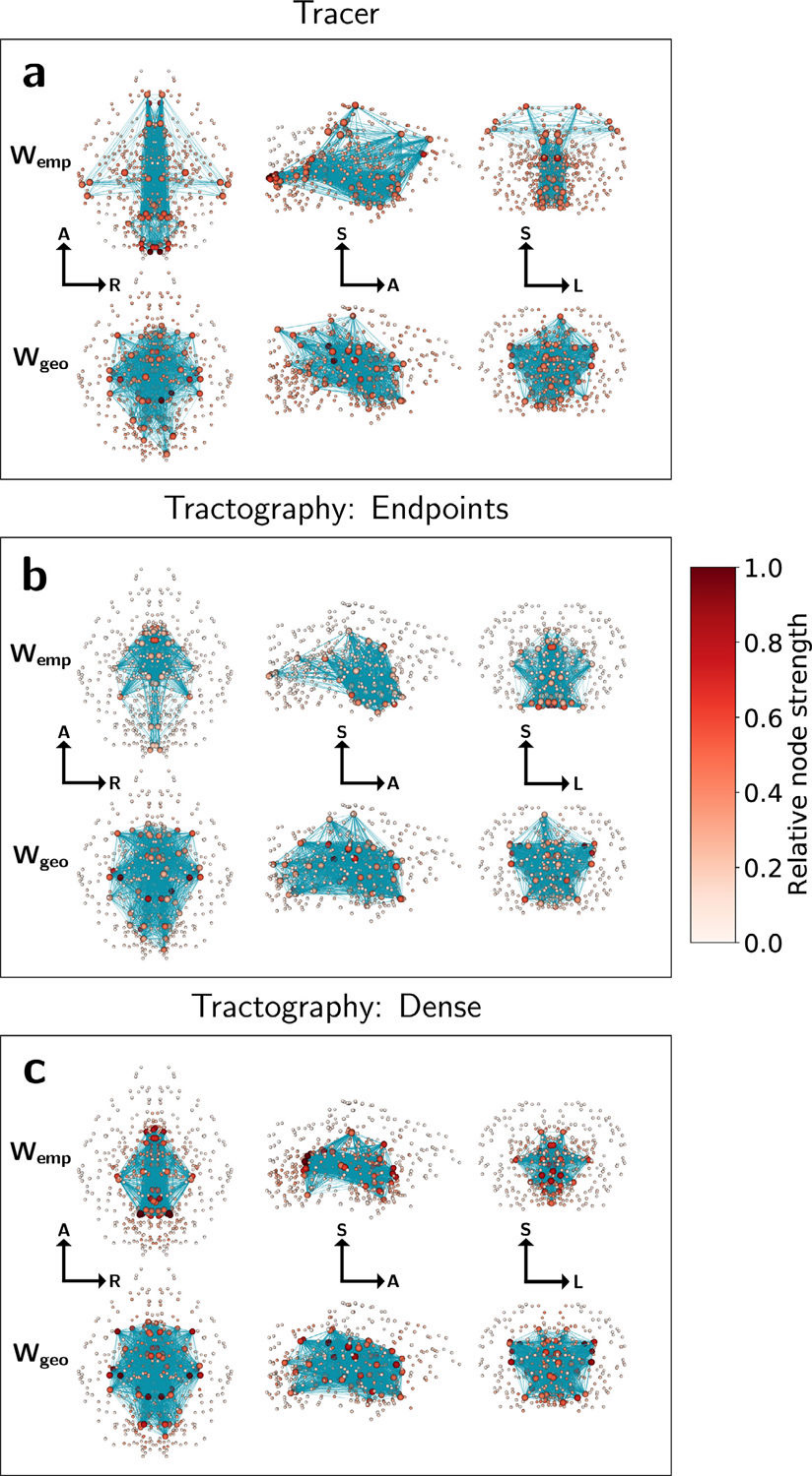
Visualization of network structure for (a) tracer, (b) endpoint, and (c) dense graphs in physical coordinates. Spheres represent the center of mass of each node, colored by relative strength. Edges between the top 15% strongest “hub” nodes (larger spheres) are shown as teal lines. Graphs are visualized along the axial (left), sagittal (middle), and coronal (right) planes with labeled orientations: A = anterior, S = superior, R = right, L = left. Tractography node strengths are calculated as averages across 5 datasets. Geometric surrogate graphs are single representative samples.

Previous work with human tractography datasets (Roberts et al., 2016) has revealed that the human brain places its strongest nodes further into geometrically peripheral regions than would be expected by weight-distance effects alone. In Fig. 6, we extend this analysis into the mouse brain and compare physical hub organization using node strength as a centrality measure. Hub node locations are visualized in physical coordinates for all empirical and geometric surrogate graphs. Hub nodes are identified as the top 15% of nodes for each graph by node strength and are visualized as large spheres. The remaining bottom 85% of nodes by strength are identified with smaller spheres. Edges between hub nodes are colored teal. For visual clarity, the remaining edges have been omitted.

Through visual comparison of empirical and geometric surrogate tracer graphs, we find the expected result that the mouse brain network as measured with tracer data places its hub nodes further towards the periphery of the brain than would be predicted by geometry alone, with the strongest nodes located across the isocortex, medulla, and inferior hypothalamus. Empirical tractography graphs, however, place the strongest nodes deeper towards the center of the brain compared to the tracer graph, with hubs organized into a ball-like structure comparable to their corresponding geometric surrogates. Table 2 shows the percent of total hub node strength located within select major brain divisions for each network construction method. 17% of the total tracer hub strength was located along the isocortex, while no isocortex nodes in either of the tractography graphs were identified as hubs. Tractography graphs likewise underestimated hub strength in the medulla and overestimated hub strength in the midbrain and thalamus, particularly for the dense node assignment strategy. Overall, only 11 individual structures were co-identified as hubs between the tracer and both tractography methods.

In the case of dense node assignment, the tendency to cluster hub nodes near the center of the brain appears even more pronounced than would be predicted by geometric surrogate graphs with the same weight-distance relationship. These results suggest a strong geometric determination in the organization of hub nodes in tractography above and beyond the geometric relationship expected from tracer data.

**Fig. 7.**
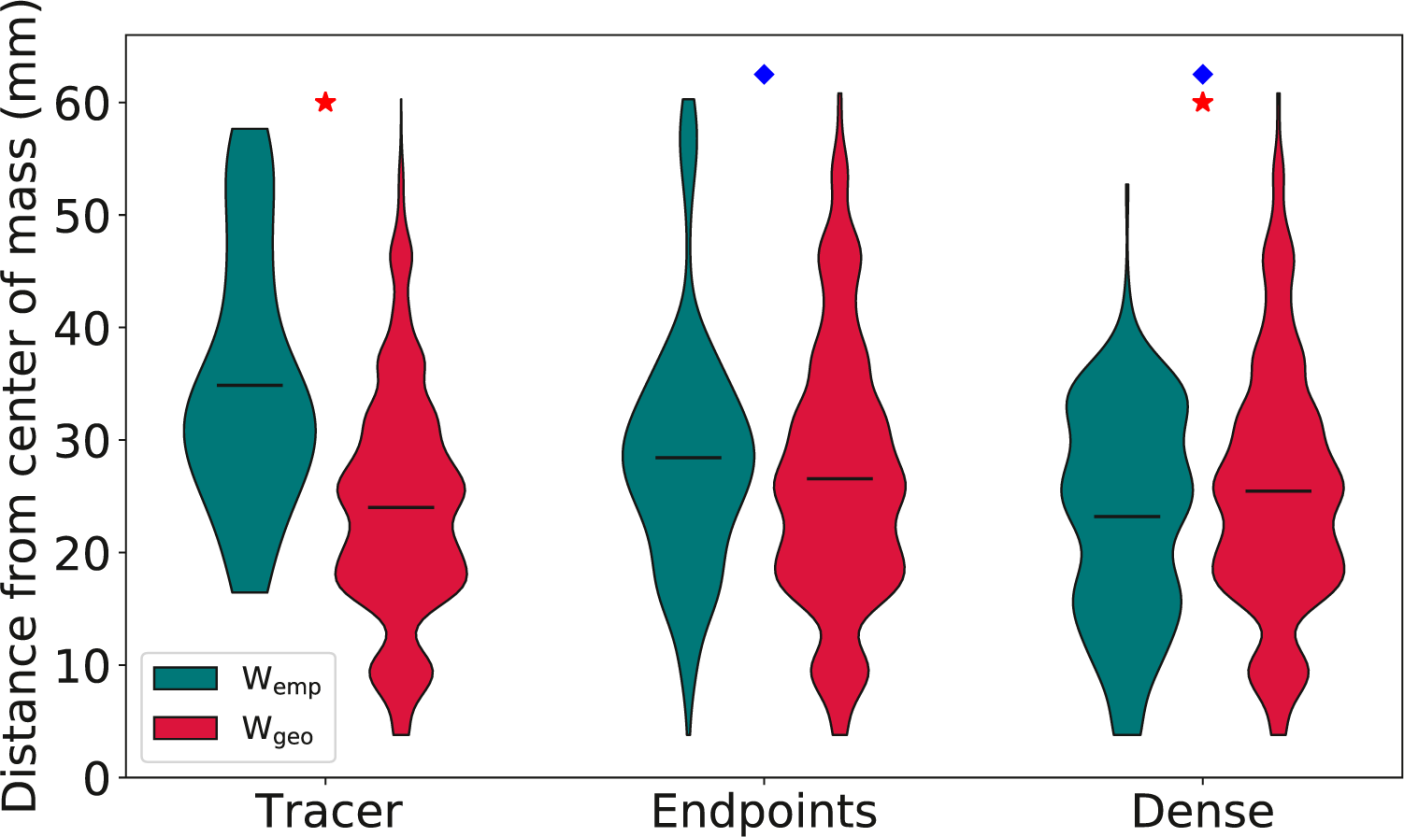
Violin plots showing the distribution of distances between the center of mass of individual hub nodes and the center of mass of the brain. Horizontal bars indicate the means of each distribution. Distributions represent per-node values across all datasets. *W*_geo_ data are taken from an ensemble of 100 random graphs per network construction method. Red stars indicate statistical significance (*p* < 0.01) in the difference between *W*_emp_ and *W*_geo_ data, and blue diamonds indicate statistical significance (*p* < 0.01) in the difference between empirical tracer and tractography data. *p*-values were calculated using Tukey’s range test.

The geometric centrality of hub nodes is further quantified in Fig. 7, which shows the mean distance between hub nodes and the center of mass of the brain for all empirical and geometric surrogate graphs. As visualized in Fig. 6, the tracer empirical graph places its hubs further from the center of mass than its corresponding geometric surrogates, while dense tractography not only places its hubs closer to the center of the brain than the tracer graph does, but also places its hubs slightly more central than its own geometric surrogates. Endpoint tractography graphs also place their hub nodes more geometrically central overall than the tracer graph, with distances from the center of mass comparable to their geometric surrogates. However, Fig. 6 shows that the endpoint graphs are better able than dense graphs to capture some of the more peripheral hubs along the inferior hypothalamus.

**Fig. 8.**
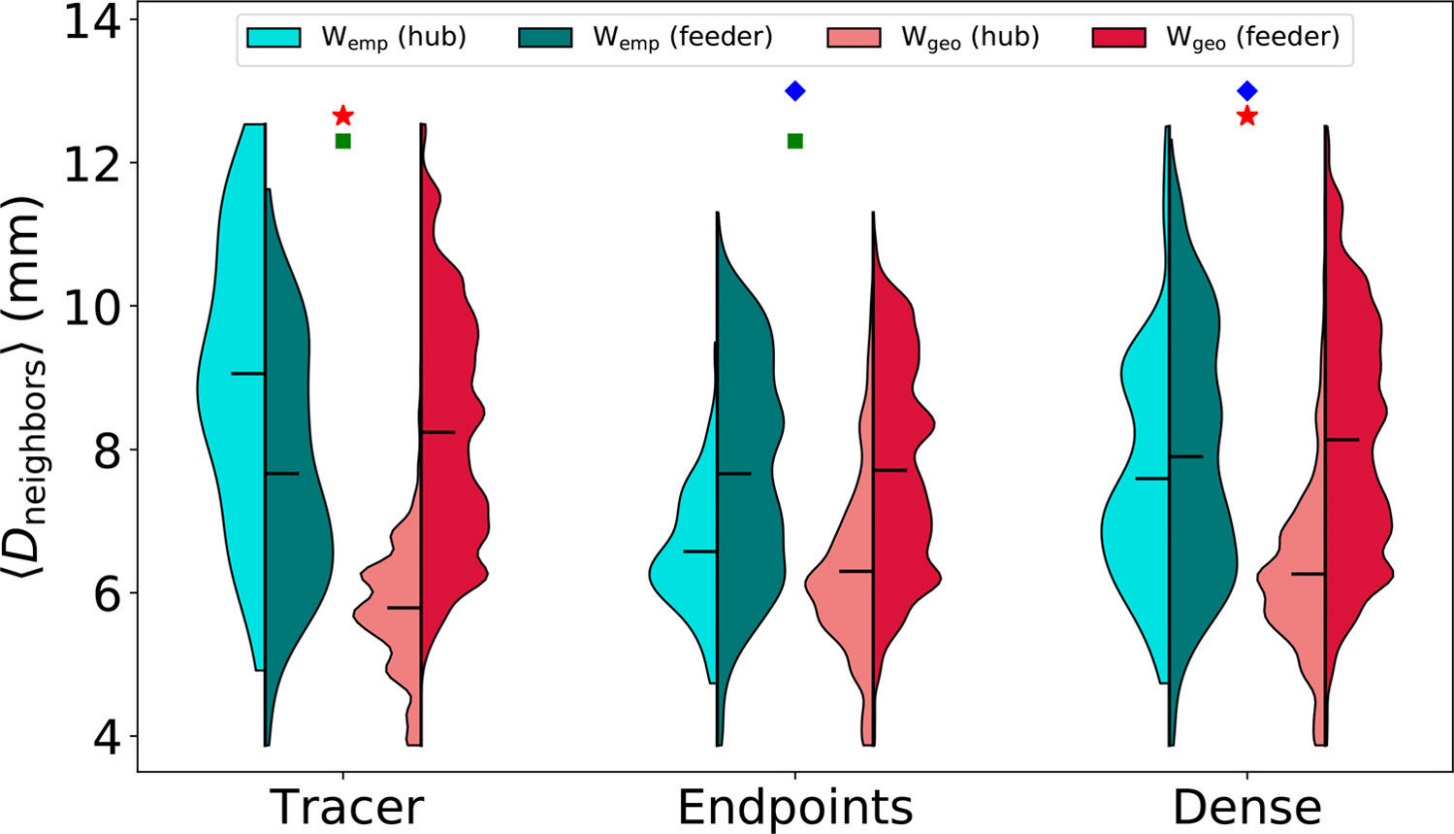
Violin plots showing distributions of the average fiber distance to each node’s neighbors, split into hub (top 15%) and feeder (bottom 85%) nodes defined using eigenvector centrality. Distributions from empirical graphs are shown in blue colors, and distributions from geometric surrogate graphs are shown in red colors. Lighter shades of each color indicate hub nodes and darker shades indicate feeder nodes. Horizontal bars indicate the means of each distribution. Distributions represent per-node values across all datasets. Green squares indicate statistical significance (*p* < 0. 01) in the difference between empirical hub and feeder distributions. Red stars indicate statistical significance (*p* < 0. 01) in the difference between *W*_emp_ and *W*_geo_ hub values. Blue diamonds indicate statistical significance (*p* < 0. 01) in the difference between empirical tracer and tractography hub values. *p*-values were calculated using Tukey’s range test.

In addition to the participation coefficient and node strength, hubs were identified and characterized using their eigenvector centrality (EC), a robust measure of relative node importance calculated by taking the elements of the leading eigenvector of the connectivity matrix (Newman, 2004). Each node’s EC is related to the weight of the connections to its neighbors, such that a node could acquire a high EC either by having a large number of very weak connections or by having a small number of very strong connections. Figure 8 shows distributions of the average fiber distance to each node’s neighbors ⟨*D*_neighbors_⟩ for all empirical and geometric surrogate graphs. Distributions are split into hub (top 15%) and “feeder” (bottom 85%) nodes defined using EC. Nodes with a low ⟨*D*_neighbors_⟩ imply physical, geometric centrality with respect to their neighbors, and nodes with a high EC imply high topological centrality and node importance.

Geometric surrogate graphs from all network construction methods have much lower mean ⟨*D*_neighbors_⟩ for hub nodes than for feeder nodes, meaning they predict the most topologically central hub nodes to also be the most geometrically central. The empirical tracer graph predicts the opposite relationship: not only are tracer hub nodes located further from their neighbors than predicted by geometry, they are also less geometrically central than the remaining feeder nodes, reflecting their peripheral placement seen in Figs. 6 and 7.

This effect is not observed in any of the empirical tractography graphs. The dense tractography graphs predict ⟨*D*_neighbors_⟩ values for their hub nodes more similar to those from the tracer graph, but the distributions from both tractography approaches are much more similar to those from their corresponding geometric surrogates than the empirical tracer distributions are to theirs. Particularly, both empirical tractography graphs have lower mean ⟨*D*_neighbors_⟩ values for hub nodes than for feeder nodes, the reverse of the relationship seen in the tracer graph. This once again indicates a strong geometric bias in the placement of topologically important nodes in tractography graphs.

### 3.4. Comparison of network efficiency

**Fig. 9.**
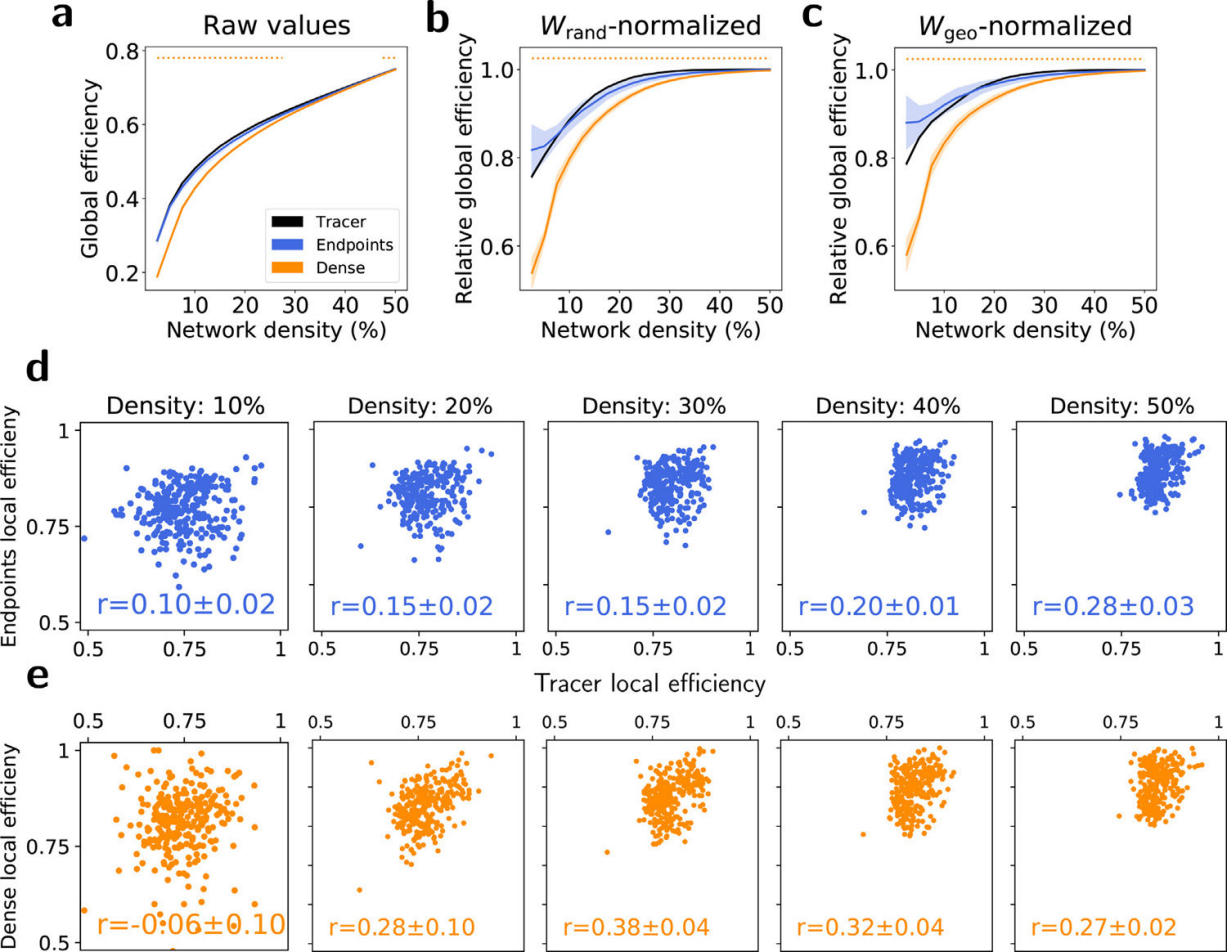
Efficiency. (a–c) Global efficiencies for each method as a function of network density. Shaded regions represent 1 standard deviation across 5 tractography datasets. (a) Raw global efficiency values for all empirical graphs. (b) Global efficiencies for all empirical graphs randomized against the mean value from their corresponding random surrogates. (c) Global efficiencies for all empirical graphs randomized against the mean value from their corresponding geometric surrogates. The widths of the horizontal lines at the top of (a–c) indicate the range of network densities with statistical significance (*p* < 0. 01) in the difference between tracer and tractography values for each tractography method, calculated with (a) Tukey’s range test and (b–c) a permutation test. (d–e) Scatterplots showing local efficiencies calculated with empirical tracer and (d) endpoint and (e) dense tractography graphs across a range of network densities. *r* values indicate Pearson correlations.

While analysis of modular structure and related metrics describe aspects of network segregation, the global and local efficiencies are metrics of integration for unweighted networks. Local efficiency is calculated as the average inverse shortest path length between a node and all of its neighbors, while global efficiency is the average inverse shortest path length between all pairs of nodes in a network. Accordingly, networks with high global efficiencies are able to efficiently communicate information across different regions. Raw global efficiency values are shown in Fig. 9a. As the underlying weighted graphs are nearly fully connected for all methods, binary efficiencies are characterized as a function of network density after thresholding low-weight edges. Global efficiencies normalized by the values from *W*_rand_- and *W*_geo_-surrogate graphs are shown in Fig. 9b–c, respectively. Across all threshold levels and normalizations, global efficiencies from endpoint tractography provided a good match to those from the tracer model. Dense tractography significantly underestimated global efficiency at all densities, even after normalization with the values from its geometric surrogates, which suggests that dense tractography underestimates the role of geometry in network integration relative to the tracer model. Figure 9d–e shows scatterplots demonstrating the relationship of per-node local efficiencies between tractography- and tracer-derived networks across multiple densities. Pearson correlations were weak across both methods and all densities, indicating that while endpoint tractography performs well in estimating global efficiency, neither tractography method is able to accurately predict the local efficiency of individual nodes. Plots comparing the additional binary topological properties of clustering coefficient and mean path length are shown in Supporting Information Figures S4–S5.

### 3.5. Validation with independent tracer measurements

**Fig. 10.**
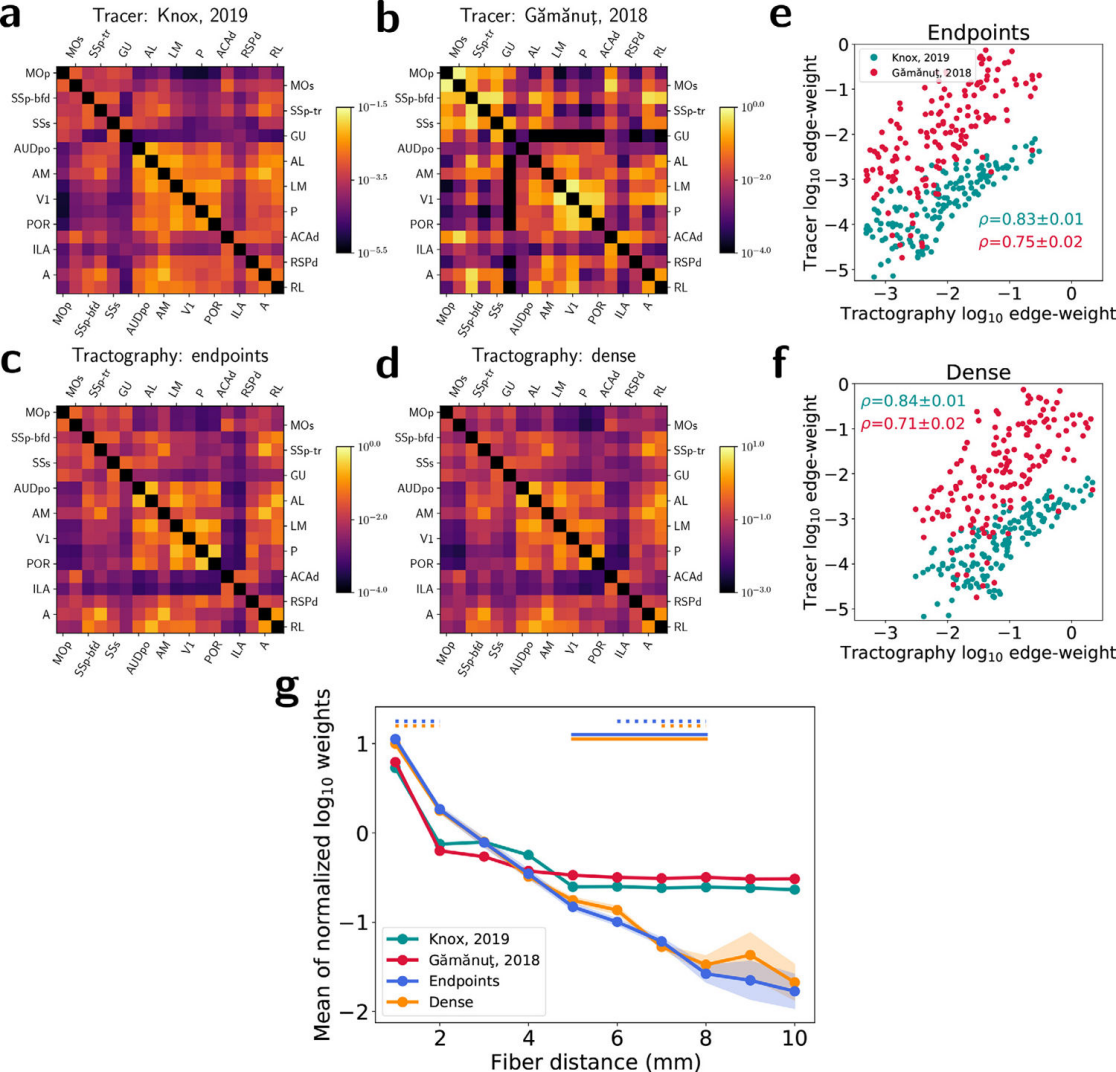
Comparison to empirical, retrograde tracer data in the cortex from Gămănuţ, 2018. (a–d) Cortical connectivity matrices for (a) the Knox, 2019 tracer model, (b) the Gămănuţ, 2018 empirical data, (c) endpoint tractography, and (d) dense tractography. (e–f) Scatterplots showing correlations between (e) endpoint and (f) dense tractography edge weights and values from the two tracer graphs. *ρ* values indicate Spearman correlation coefficients with standard deviations calculated across 5 tractography datasets. (g) Normalized weight-distance relationships. Circles represent the average log-weight z-scores for each method within 1 mm fiber distance bins. Shaded regions represent 1 standard deviation across 5 tractography datasets. The widths of the dotted and solid horizontal lines at the top of the figure indicate the range of fiber distance bins with statistically significant differences (*p* < 0. 01) between Knox, 2019 (dotted) and Gămănuţ, 2018 (solid) tracer values and tractography values for each tractography method, calculated using a one-sample *t*-test after correcting for multiple comparisons.

For independent validation of the whole-brain network analysis results from the computational tracer model from Knox et al., tractography edge-weights were also compared to ipsilateral cortical connectivity measurements published in Gămănuţ et al. (2018). Figs. 10a–d show connectivity matrices of 18 cortical regions from both tracer datasets and tractography methods. Scatterplots between tractography and tracer edge-weights are shown for endpoints and dense tractography in Fig. 10e–f, respectively. For both node assignment methods, Spearman correlations between tractography and tracer weights are significantly higher with the Knox et al. model than the Gămănuţ et al. data. Fig. 10g shows the mean relationship between edge weights and fiber distance after the log-weight distributions were normalized to a mean of 0 and standard deviation of 1. Even though the overall range of distances is shorter between ipsilateral cortical nodes than across the whole brain, the tractography methods both still demonstrate a significant underestimation of long-range connectivity relative to empirical tract-tracing measurements in the cortex, which is consistent with the resulting geometric bias in network properties found through comparison to computational tracer-derived connectivity estimates in the whole brain.

## 4. Discussion

Across nearly all metrics explored in this study, we find that the topological properties of tractography-derived graphs are much more influenced by spatial embedding than would be predicted by the more accurate role of spatial embedding represented by the tracer model. Tractography graphs underestimate connectivity weights at long distances, leading to a conflation of topological and geometric centrality that biases the estimated modular structure and the architecture of hub subnetworks. These results serve as an important reminder for consideration in future tractography studies: given that many properties of the true brain network can be reasonably predicted strictly by spatial embedding, tractography methods development and validation efforts should be targeted towards the ability to predict network properties *beyond* a geometric baseline. While we expect that methodological geometric bias plays a similar role in human tractography networks, some studies have shown encouraging results. For example, Roberts et al. (2016) demonstrated that empirical human tractography graphs exhibit a more peripheral hub network structure than predicted by their corresponding geometric surrogate graphs, similar to our tracer results in Fig. 6 and in contrast to what we observed with tractography networks. Nevertheless, conclusions from human tractography networks cannot be verified with additional ground-truth imaging, and caution should be taken when interpreting tractography-derived brain networks in all species, particularly for metrics which rely more on long-range connections.

One aim of this work was to explore differences in tractography network structure resulting from the streamline node-assignment strategy. Ultimately, we found our results were largely independent of the specific node-assignment approach. The geometric bias in tractography was not mitigated by either method; despite small differences, network characteristics of both tractography graphs were far more similar to each other than either of them were to characteristics of the tracer graph. While endpoint tractography might represent a more physically intuitive model of brain connectivity, the comparable performance of dense tractography reflects the ambiguous physical definition of tractography streamlines. Particularly at this spatial resolution and in the absence of stronger anatomical regularization, tractography streamlines strictly represent potential probabilistic pathways of white matter fibers that are consistent with symmetric diffusion data. When streamlines are made to terminate under reasonably enforced constraints such as on fODF magnitude or streamline curvature, this enforces a penalty on unrealistic fiber geometries or the use of lower-confidence diffusion data, but does not yield a physical sense of the actual origin or termination points of the underlying neuronal fibers. This compromises the intuitive appeal of “endpoint” streamline node assignment as used in this study. In fact, without additional constraints, we expect the endpoint locations of any given streamline to be more noisy and erroneous as streamline length increases due to the fiber orientation errors accumulated at each step in the tracking process, an inherent tradeoff that exists even with more sophisticated forms of anatomic regularization. With dense tractography, the effective signal to noise ratio for connectivity similarly falls off for points further away from the seed location, but since a single streamline is allowed to contribute to the connectivity estimate between multiple node-pairs at varying distances, a greater proportion of points from each streamline contribute connectivity estimates that are less noisy than those from the endpoints. Dense tractography also serves as a potentially more physically meaningful match to the tracer data used in this study, as connectivity values in the Knox et al. model are derived from segmented projection volumes of a viral tracer that fluoresces along the entire length of any given neural projection.

For the data used in this study, we took the approach of seeding only from the gray matter to avoid known tract-length connectivity biases (Girard et al., 2014; Jeurissen et al., 2019), and normalized the streamline counts by the volumes of each node-pair in order to better match the normalized connection density metric used in the Knox et al. model. Since our data were acquired with a single, relatively low b-value, we opted against the use of more advanced streamline quantitation algorithms such as SIFT2 (Smith et al., 2015). This also provides a more direct comparison between our results and those of similar studies that have used the Allen tracer data to benchmark tractography performance without reference to the role of spatial embedding (Aydogan et al., 2018; Calabrese et al., 2015; Chen et al., 2015). Even with these relatively simple post-processing approaches, the results of this study serve to echo recent tractography validation reviews that suggest that the future of tractography connectomics hinges on the incorporation of more advanced anatomical and microstructural priors to tractography pipelines in order to address geometric and other biases and make streamlines more quantitative and physically meaningful (Maier-Hein et al., 2017; Schilling et al., 2019). Network analysis with geometric surrogate graphs can be an important tool to evaluate such quantitative tractography pipelines in the future. A recent study by Girard et al. (2020) rigorously benchmarked 15 tractography algorithms and a number of regularization approaches such as the “anatomically constrained tractography” framework (Smith et al., 2012) and the SIFT2 post-processing algorithm (Smith et al., 2015) against tracer data in the macaque cortex, though it did not present analysis of downstream network measures. Our future work will similarly explore the adoption of more advanced tractography and regularization approaches in the mouse brain, where whole-brain tracer data are more readily available. Benchmarking these approaches with the use of geometric surrogate graphs will allow for a deeper understanding of the value of existing quantitative strategies designed to mitigate tractography biases.

The results in this study rely on the assumption of the tracer data as a ground truth representation of the underlying mesoscale mouse brain network architecture. While tracer data is certainly ideal in many respects for the benchmarking of diffusion tractography, there are also limitations to this assumption. Both anterograde and retrograde tracer studies produce inherently directed graphs, whereas tractography is based on inherently symmetric diffusion measurements and produces undirected graphs. This requires the use of directional symmetry enforcement for fair comparison that may alter the underlying network properties represented by the tracer data. Besides any biases in the imaging and registration process itself, the Knox et al. model relies on computational estimates of connectivity based on segmented volume fractions of underlying tracer experiments that may span multiple different gray-matter regions. Accordingly, the Knox et al. model itself is only an estimate that may carry its own biases of the true underlying density of neurons connecting each region pair. For this reason, we are encouraged by the validation of our tractography results against the more empirical measurements of neuronal connectivity from the retrograde tract-tracing experiments published by Gămănuţ et al. (Fig. 10). Correlations between tractography and tracer edge-weights are even lower for the Gămănuţ et al. data than for the Knox et al. model, while tractography shows a comparably dramatic falloff in weight with distance relative to both tracer datasets, suggesting that our overall conclusion that tractography graphs are more determined by geometry than tracer graphs would persist if empirical measurements similar to those from the Gămănuţ et al. study were available across the whole brain.

## 5. Conclusion

We used geometric surrogate graphs to explore the role of spatial embedding in the topological properties of the mouse structural brain network measured by neural tracer imaging and diffusion MRI tractography. We found that spatial embedding played a considerably larger role in the topology of tractography networks than tracer networks. Tractography approaches underestimate long-range connectivity, which leads to geometric biases in the estimated modular structure and placement of high-strength hub nodes. Our results demonstrate the caution required in the interpretation of tractography-derived network measurements that rely on long-range connections and motivate additional geometric consideration in the design of future tractography validation studies.

## Supplementary Material

1

## Figures and Tables

**Table 1:** Percent agreement in consensus node-module assignment.

	Tracer	Endpoints	Dense
Tractography vs. tracer	—	54	54
Empirical vs. geometric surrogates	53	66	67

**Table 2:** Percent of total hub node strength contained in select major brain divisions.

Region	Tracer	Endpoints	Dense
Hypothalamus	29	57	22
Medulla	19	9	7
Isocortex	17	0	0
Midbrain	10	12	22
Thalamus	9	9	31
