# Supplementary material for: The role of spatial embedding in mouse brain networks constructed from diffusion tractography and tracer injections
Source: Neuroimage. Author manuscript; Available in PMC 2021 Dec 1. (PMC8611903; doi:10.1016/j.neuroimage.2021.118576)
Supplement: 1 [file NIHMS1755362-supplement-1.pdf]

The role of spatial embedding in mouse brain networks constructed from diffusion tractography and tracer injections

Supporting Information

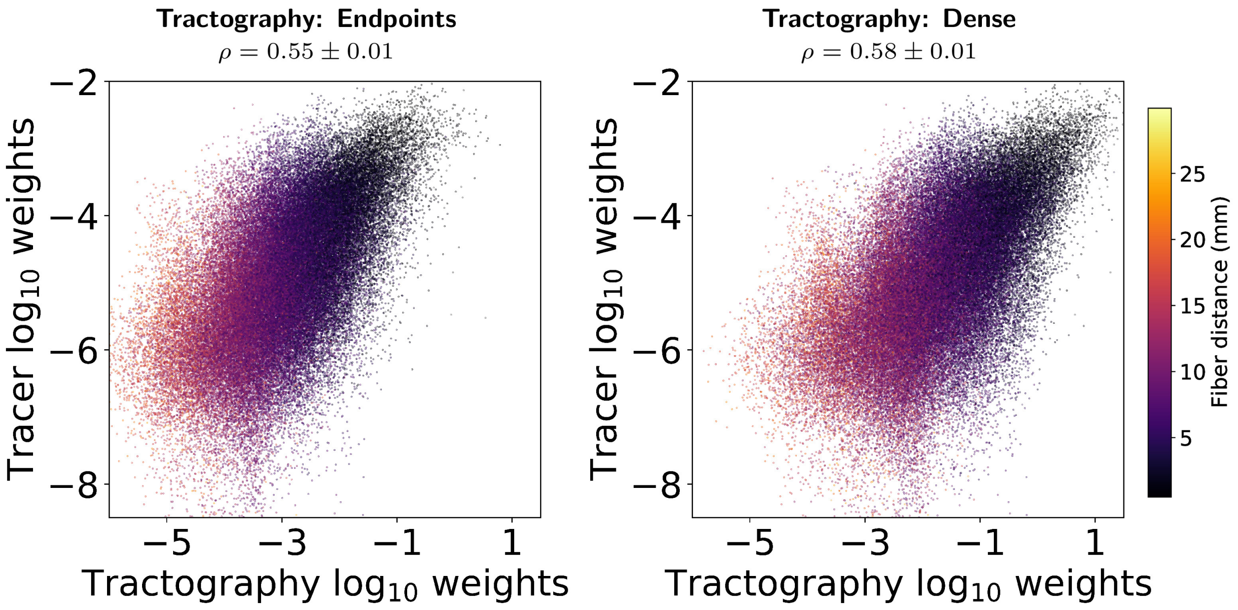

Figure S1: Scatterplots of the log-weights for tracer vs. tractography connectivity matrices. Color indicates fiber distance. Tractography weight values represent averages across 5 datasets. ρ values indicate Spearman rank correlation coefficients, with standard deviations across 5 tractography datasets.

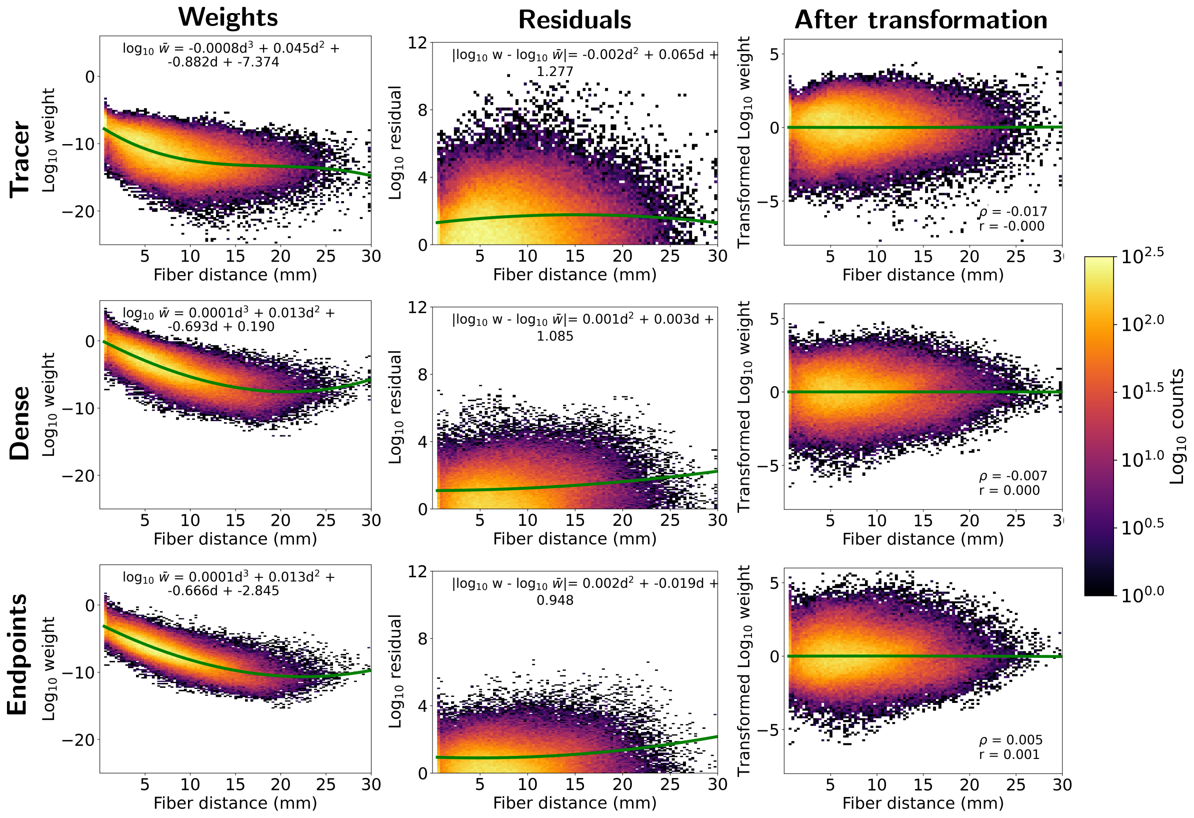

Figure S2: Distance curves for weights (left) and residuals (center) for all network construction methods. The resulting weight-distance relationships after transformation are shown on the right with corresponding linear fits (green line). r and ρ values indicate Pearson and Spearman correlation coefficients, respectively. Counts of individual edges within weight-distance bins are represented as a heatmap on a log color scale. Polynomial fits are shown as green curves. Tractography values represent averages across 5 datasets.

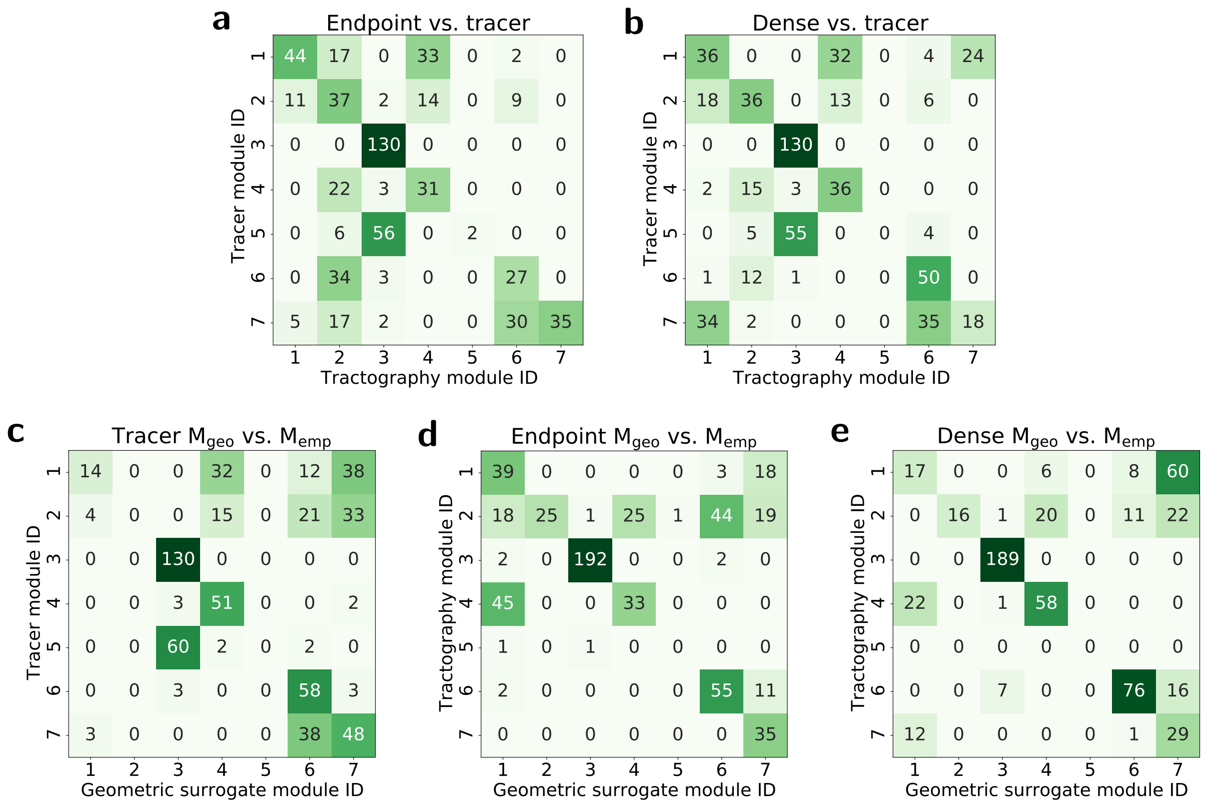

Figure S3: Confusion matrices for module assignment. Module IDs correspond to those identified with the colorbar in Figure 3. (a–b) Module assignments between empirical tracer and empirical (a) endpoints and (b) dense tractography graphs. (c–e) Assignments between empirical (c) tracer, (d) endpoint, and (e) dense graphs and those from their corresponding geometric surrogate graphs. Values represent consensus assignments across 5 tractography datasets and an ensemble of 100 geometric surrogate graphs.

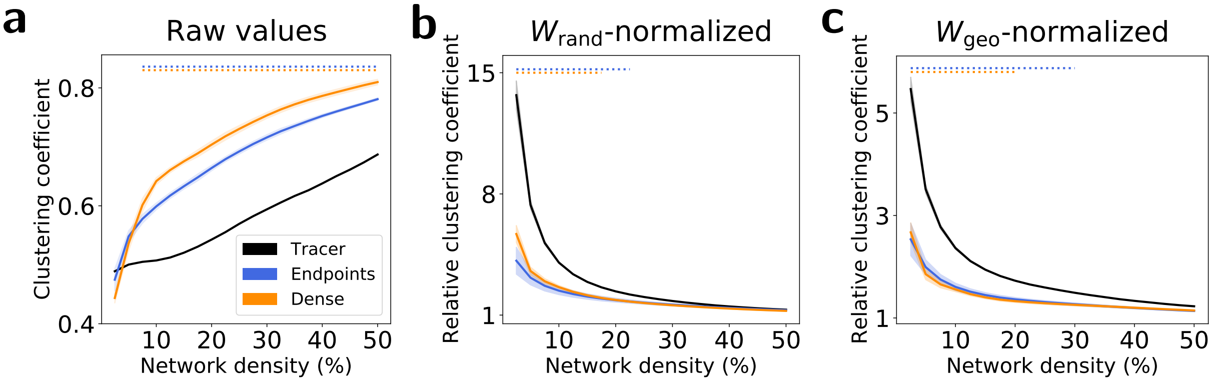

Figure S4: Mean binary clustering coefficients for each method as a function of network density. Shaded regions represent 1 standard deviation across 5 tractography datasets and ensembles of 100 geometric and random surrogate graphs. (a) Raw mean clustering coefficient values for all empirical graphs. (b) Mean clustering coefficients for all empirical graphs randomized against the mean value from their corresponding random surrogates. (c) Mean clustering coefficients for all empirical graphs randomized against the mean value from their corresponding geometric surrogates. The widths of the horizontal lines at the top of the figures indicate the range of network densities with statistical significance ($p < 0.01$) in the difference between tracer and tractography values for each tractography method, calculated with (a) Tukey's range test and (b–c) a permutation test.

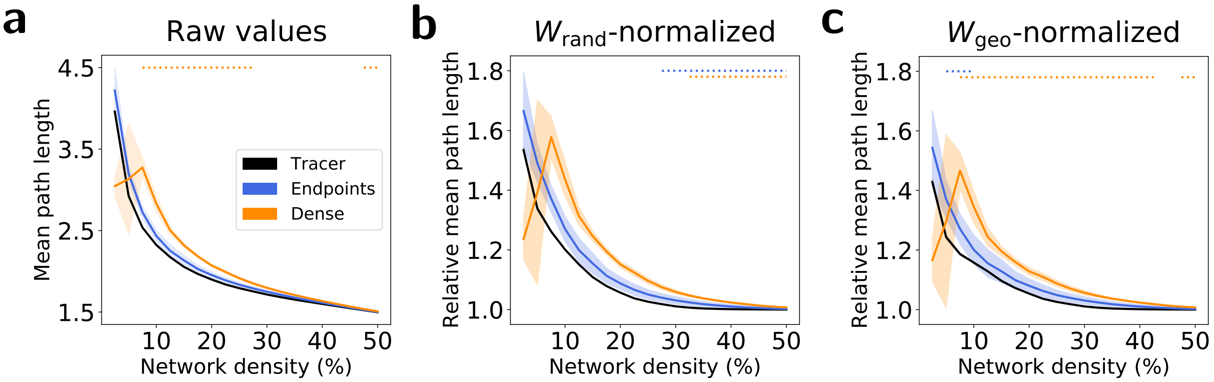

Figure S5: Mean binary path lengths for each method as a function of network density. Shaded regions represent 1 standard deviation across 5 tractography datasets and ensembles of 100 geometric and random surrogate graphs. (a) Raw mean path length values for all empirical graphs. (b) Mean path lengths for all empirical graphs randomized against the mean value from their corresponding random surrogates. (c) Mean path lengths for all empirical graphs randomized against the mean value from their corresponding geometric surrogates. The widths of the horizontal lines at the top of the figures indicate the range of network densities with statistical significance ($p < 0.01$) in the difference between tracer and tractography values for each tractography method, calculated with (a) Tukey's range test and (b–c) a permutation test.

Table S1: Parcellation structure information from the Allen Mouse Brain Atlas.

Abbreviation	Allen structure ID	Full structure name	Major brain division
FRP	184	Frontal pole, cerebral cortex	Isocortex
MOp	985	Primary motor area	Isocortex
MOs	993	Secondary motor area	Isocortex
SSp-n	353	Primary somatosensory area, nose	Isocortex
SSp-bfd	329	Primary somatosensory area, barrel field	Isocortex
SSp-ll	337	Primary somatosensory area, lower limb	Isocortex
SSp-m	345	Primary somatosensory area, mouth	Isocortex
SSp-ul	369	Primary somatosensory area, upper limb	Isocortex
SSp-tr	361	Primary somatosensory area, trunk	Isocortex
SSp-un	182305689	Primary somatosensory area, unassigned	Isocortex
SSs	378	Supplemental somatosensory area	Isocortex
GU	1057	Gustatory areas	Isocortex
VISC	677	Visceral area	Isocortex
AUDd	1011	Dorsal auditory area	Isocortex
AUDp	1002	Primary auditory area	Isocortex
AUDpo	1027	Posterior auditory area	Isocortex
AUDv	1018	Ventral auditory area	Isocortex
VISal	402	Anterolateral visual area	Isocortex
VISam	394	Anteromedial visual area	Isocortex
VISl	409	Lateral visual area	Isocortex
VISp	385	Primary visual area	Isocortex
VISpl	425	Posterolateral visual area	Isocortex
VISpm	533	posteromedial visual area	Isocortex
VISli	312782574	Laterointermediate area	Isocortex
VISpor	312782628	Postrhinal area	Isocortex
ACAd	39	Anterior cingulate area, dorsal part	Isocortex
ACAv	48	Anterior cingulate area, ventral part	Isocortex
PL	972	Prelimbic area	Isocortex
ILA	44	Infralimbic area	Isocortex
ORBl	723	Orbital area, lateral part	Isocortex
ORBm	731	Orbital area, medial part	Isocortex
ORBvl	746	Orbital area, ventrolateral part	Isocortex
AId	104	Agranular insular area, dorsal part	Isocortex
AIp	111	Agranular insular area, posterior part	Isocortex
AIv	119	Agranular insular area, ventral part	Isocortex
RSPagl	894	Retrosplenial area, lateral agranular part	Isocortex
RSPd	879	Retrosplenial area, dorsal part	Isocortex
RSPv	886	Retrosplenial area, ventral part	Isocortex
VISa	312782546	Anterior area	Isocortex
VISrl	417	Rostrolateral visual area	Isocortex
TEa	541	Temporal association areas	Isocortex
PERI	922	Perirhinal area	Isocortex
ECT	895	Ectorhinal area	Isocortex
MOB	507	Main olfactory bulb	Olfactory areas
AOB	151	Accessory olfactory bulb	Olfactory areas
AON	159	Anterior olfactory nucleus	Olfactory areas
TT	589	Taenia tecta	Olfactory areas
DP	814	Dorsal peduncular area	Olfactory areas
PIR	961	Piriform area	Olfactory areas
NLOT	619	Nucleus of the lateral olfactory tract	Olfactory areas
COAa	639	Cortical amygdalar area, anterior part	Olfactory areas
COAp	647	Cortical amygdalar area, posterior part	Olfactory areas
PAA	788	Piriform-amygdalar area	Olfactory areas
TR	566	Postpiriform transition area	Olfactory areas
CA1	382	Field CA1	Hippocampal formation
CA2	423	Field CA2	Hippocampal formation
CA3	463	Field CA3	Hippocampal formation
DG	726	Dentate gyrus	Hippocampal formation
FC	982	Fasciola cinerea	Hippocampal formation
IG	19	Induseum griseum	Hippocampal formation
ENTl	918	Entorhinal area, lateral part	Hippocampal formation
ENTm	926	Entorhinal area, medial part, dorsal zone	Hippocampal formation
PAR	843	Parasubiculum	Hippocampal formation
POST	1037	Postsubiculum	Hippocampal formation

Continued on next page

Continued from previous page

Abbreviation	Allen structure ID	Full structure name	Major brain division
PRE	1084	Presubiculum	Hippocampal formation
SUB	502	Subiculum	Hippocampal formation
CLA	583	Claustrum	Cortical subplate
EPd	952	Endopiriform nucleus, dorsal part	Cortical subplate
EPv	966	Endopiriform nucleus, ventral part	Cortical subplate
LA	131	Lateral amygdalar nucleus	Cortical subplate
BLA	295	Basolateral amygdalar nucleus	Cortical subplate
BMA	319	Basomedial amygdalar nucleus	Cortical subplate
PA	780	Posterior amygdalar nucleus	Cortical subplate
CP	672	Caudoputamen	Striatum
ACB	56	Nucleus accumbens	Striatum
FS	998	Fundus of striatum	Striatum
OT	754	Olfactory tubercle	Striatum
LSc	250	Lateral septal nucleus, caudal (caudodorsal) part	Striatum
LSr	258	Lateral septal nucleus, rostral (rostroventral) part	Striatum
LSv	266	Lateral septal nucleus, ventral part	Striatum
SF	310	Septofimbrial nucleus	Striatum
SH	333	Septohippocampal nucleus	Striatum
AAA	23	Anterior amygdalar area	Striatum
BA	292	Bed nucleus of the accessory olfactory tract	Striatum
CEA	536	Central amygdalar nucleus	Striatum
IA	1105	Intercalated amygdalar nucleus	Striatum
MEA	403	Medial amygdalar nucleus	Striatum
GPe	1022	Globus pallidus, external segment	Pallidum
GPi	1031	Globus pallidus, internal segment	Pallidum
SI	342	Substantia innominata	Pallidum
MA	298	Magnocellular nucleus	Pallidum
MS	564	Medial septal nucleus	Pallidum
NDB	596	Diagonal band nucleus	Pallidum
TRS	581	Triangular nucleus of septum	Pallidum
BST	351	Bed nuclei of the stria terminalis	Pallidum
BAC	287	Bed nucleus of the anterior commissure	Pallidum
VAL	629	Ventral anterior-lateral complex of the thalamus	Thalamus
VM	685	Ventral medial nucleus of the thalamus	Thalamus
VPL	718	Ventral posterolateral nucleus of the thalamus	Thalamus
VPLpc	725	Ventral posterolateral nucleus of the thalamus, parvicellular part	Thalamus
VPM	733	Ventral posteromedial nucleus of the thalamus	Thalamus
VPMpc	741	Ventral posteromedial nucleus of the thalamus, parvicellular part	Thalamus
SPFm	414	Subparafascicular nucleus, magnocellular part	Thalamus
SPFp	422	Subparafascicular nucleus, parvicellular part	Thalamus
SPA	609	Subparafascicular area	Thalamus
PP	1044	Peripeduncular nucleus	Thalamus
MG	475	Medial geniculate complex	Thalamus
LGd	170	Dorsal part of the lateral geniculate complex	Thalamus
LP	218	Lateral posterior nucleus of the thalamus	Thalamus
PO	1020	Posterior complex of the thalamus	Thalamus
POL	1029	Posterior limiting nucleus of the thalamus	Thalamus
SGN	325	Suprageniculate nucleus	Thalamus
AV	255	Anteroventral nucleus of thalamus	Thalamus
AM	127	Anteromedial nucleus	Thalamus
AD	64	Anterodorsal nucleus	Thalamus
IAM	1120	Interanteromedial nucleus of the thalamus	Thalamus
IAD	1113	Interanterodorsal nucleus of the thalamus	Thalamus
LD	155	Lateral dorsal nucleus of thalamus	Thalamus
IMD	59	Intermediodorsal nucleus of the thalamus	Thalamus
MD	362	Mediodorsal nucleus of thalamus	Thalamus
SMT	366	Submedial nucleus of the thalamus	Thalamus
PR	1077	Perireunensis nucleus	Thalamus
PVT	149	Paraventricular nucleus of the thalamus	Thalamus
PT	15	Parataenial nucleus	Thalamus
RE	181	Nucleus of reuniens	Thalamus
RH	189	Rhomboid nucleus	Thalamus
CM	599	Central medial nucleus of the thalamus	Thalamus
PCN	907	Paracentral nucleus	Thalamus
CL	575	Central lateral nucleus of the thalamus	Thalamus

Continued on next page

Continued from previous page

Abbreviation	Allen structure ID	Full structure name	Major brain division
PF	930	Parafascicular nucleus	Thalamus
RT	262	Reticular nucleus of the thalamus	Thalamus
IGL	27	Intergeniculate leaflet of the lateral geniculate complex	Thalamus
LGv	178	Ventral part of the lateral geniculate complex	Thalamus
SubG	321	Subgeniculate nucleus	Thalamus
MH	483	Medial habenula	Thalamus
LH	186	Lateral habenula	Thalamus
SO	390	Supraoptic nucleus	Hypothalamus
ASO	332	Accessory supraoptic group	Hypothalamus
PVH	38	Paraventricular hypothalamic nucleus	Hypothalamus
PVa	30	Periventricular hypothalamic nucleus, anterior part	Hypothalamus
PVi	118	Periventricular hypothalamic nucleus, intermediate part	Hypothalamus
ARH	223	Arcuate hypothalamic nucleus	Hypothalamus
ADP	72	Anterodorsal preoptic nucleus	Hypothalamus
AVP	263	Anteroventral preoptic nucleus	Hypothalamus
AVPV	272	Anteroventral periventricular nucleus	Hypothalamus
DMH	830	Dorsomedial nucleus of the hypothalamus	Hypothalamus
MEPO	452	Median preoptic nucleus	Hypothalamus
MPO	523	Medial preoptic area	Hypothalamus
PD	914	Posterodorsal preoptic nucleus	Hypothalamus
PS	1109	Parastrial nucleus	Hypothalamus
PVp	126	Periventricular hypothalamic nucleus, posterior part	Hypothalamus
PVpo	133	Periventricular hypothalamic nucleus, preoptic part	Hypothalamus
SBPV	347	Subparaventricular zone	Hypothalamus
SCH	286	Suprachiasmatic nucleus	Hypothalamus
SFO	338	Subfornical organ	Hypothalamus
VLPO	689	Ventrolateral preoptic nucleus	Hypothalamus
AHN	88	Anterior hypothalamic nucleus	Hypothalamus
LM	210	Lateral mammillary nucleus	Hypothalamus
MM	491	Medial mammillary nucleus	Hypothalamus
SUM	525	Supramammillary nucleus	Hypothalamus
TMd	1126	Tuberomammillary nucleus, dorsal part	Hypothalamus
TMv	1	Tuberomammillary nucleus, ventral part	Hypothalamus
MPN	515	Medial preoptic nucleus	Hypothalamus
PMd	980	Dorsal premammillary nucleus	Hypothalamus
PMv	1004	Ventral premammillary nucleus	Hypothalamus
PVHd	63	Paraventricular hypothalamic nucleus, descending division	Hypothalamus
VMH	693	Ventromedial hypothalamic nucleus	Hypothalamus
PH	946	Posterior hypothalamic nucleus	Hypothalamus
LHA	194	Lateral hypothalamic area	Hypothalamus
LPO	226	Lateral preoptic area	Hypothalamus
PST	356	Preparasubthalamic nucleus	Hypothalamus
PSTN	364	Parasubthalamic nucleus	Hypothalamus
RCH	173	Retrochiasmatic area	Hypothalamus
STN	470	Subthalamic nucleus	Hypothalamus
TU	614	Tuberal nucleus	Hypothalamus
ZI	797	Zona incerta	Hypothalamus
SCs	302	Superior colliculus, sensory related	Midbrain
IC	4	Inferior colliculus	Midbrain
NB	580	Nucleus of the brachium of the inferior colliculus	Midbrain
SAG	271	Nucleus sagulum	Midbrain
PBG	874	Parabigeminal nucleus	Midbrain
MEV	460	Midbrain trigeminal nucleus	Midbrain
SNr	381	Substantia nigra, reticular part	Midbrain
VTA	749	Ventral tegmental area	Midbrain
RR	246	Midbrain reticular nucleus, retrorubral area	Midbrain
MRN	128	Midbrain reticular nucleus	Midbrain
SCm	294	Superior colliculus, motor related	Midbrain
PAG	795	Periaqueductal gray	Midbrain
APN	215	Anterior pretectal nucleus	Midbrain
MPT	531	Medial pretectal area	Midbrain
NOT	628	Nucleus of the optic tract	Midbrain
NPC	634	Nucleus of the posterior commissure	Midbrain
OP	706	Olivary pretectal nucleus	Midbrain
PPT	1061	Posterior pretectal nucleus	Midbrain

Continued on next page

Continued from previous page

Abbreviation	Allen structure ID	Full structure name	Major brain division
CUN	616	Cuneiform nucleus	Midbrain
RN	214	Red nucleus	Midbrain
III	35	Oculomotor nucleus	Midbrain
IV	115	Trochlear nucleus	Midbrain
VTN	757	Ventral tegmental nucleus	Midbrain
AT	231	Anterior tegmental nucleus	Midbrain
LT	66	Lateral terminal nucleus of the accessory optic tract	Midbrain
SNc	374	Substantia nigra, compact part	Midbrain
PPN	1052	Pedunculopontine nucleus	Midbrain
IF	12	Interfascicular nucleus raphe	Midbrain
IPN	100	Interpeduncular nucleus	Midbrain
RL	197	Rostral linear nucleus raphe	Midbrain
CLI	591	Central linear nucleus raphe	Midbrain
DR	872	Dorsal nucleus raphe	Midbrain
NLL	612	Nucleus of the lateral lemniscus	Pons
PSV	7	Principal sensory nucleus of the trigeminal	Pons
PB	867	Parabrachial nucleus	Pons
SOC	398	Superior olivary complex	Pons
B	280	Barrington's nucleus	Pons
DTN	880	Dorsal tegmental nucleus	Pons
PCG	898	Pontine central gray	Pons
PG	931	Pontine gray	Pons
PRNc	1093	Pontine reticular nucleus, caudal part	Pons
SG	318	Supragenual nucleus	Pons
SUT	534	Supratrigeminal nucleus	Pons
TRN	574	Tegmental reticular nucleus	Pons
V	621	Motor nucleus of trigeminal	Pons
CS	679	Superior central nucleus raphe	Pons
LC	147	Locus ceruleus	Pons
LDT	162	Laterodorsal tegmental nucleus	Pons
NI	604	Nucleus incertus	Pons
PRNr	146	Pontine reticular nucleus	Pons
RPO	238	Nucleus raphe pontis	Pons
SLC	350	Subceruleus nucleus	Pons
SLD	358	Sublaterodorsal nucleus	Pons
AP	207	Area postrema	Medulla
DCO	96	Dorsal cochlear nucleus	Medulla
VCO	101	Ventral cochlear nucleus	Medulla
CU	711	Cuneate nucleus	Medulla
GR	1039	Gracile nucleus	Medulla
ECU	903	External cuneate nucleus	Medulla
NTB	642	Nucleus of the trapezoid body	Medulla
NTS	651	Nucleus of the solitary tract	Medulla
SPVC	429	Spinal nucleus of the trigeminal, caudal part	Medulla
SPVI	437	Spinal nucleus of the trigeminal, interpolar part	Medulla
SPVO	445	Spinal nucleus of the trigeminal, oral part	Medulla
VI	653	Abducens nucleus	Medulla
VII	661	Facial motor nucleus	Medulla
ACVII	576	Accessory facial motor nucleus	Medulla
AMB	135	Nucleus ambiguus	Medulla
DMX	839	Dorsal motor nucleus of the vagus nerve	Medulla
GRN	1048	Gigantocellular reticular nucleus	Medulla
ICB	372	Infracerebellar nucleus	Medulla
IO	83	Inferior olivary complex	Medulla
IRN	136	Intermediate reticular nucleus	Medulla
ISN	106	Inferior salivatory nucleus	Medulla
LIN	203	Linear nucleus of the medulla	Medulla
LRN	235	Lateral reticular nucleus	Medulla
MARN	307	Magnocellular reticular nucleus	Medulla
MDRNd	1098	Medullary reticular nucleus, dorsal part	Medulla
MDRNv	1107	Medullary reticular nucleus, ventral part	Medulla
PARN	852	Parvicellular reticular nucleus	Medulla
PAS	859	Parasolitary nucleus	Medulla
PGRNd	970	Paragigantocellular reticular nucleus, dorsal part	Medulla
PGRNl	978	Paragigantocellular reticular nucleus, lateral part	Medulla

Continued on next page

Continued from previous page

Abbreviation	Allen structure ID	Full structure name	Major brain division
NR	177	Nucleus of Roller	Medulla
PRP	169	Nucleus prepositus	Medulla
PPY	1069	Parapyramidal nucleus	Medulla
LAV	209	Lateral vestibular nucleus	Medulla
MV	202	Medial vestibular nucleus	Medulla
SPIV	225	Spinal vestibular nucleus	Medulla
SUV	217	Superior vestibular nucleus	Medulla
x	765	Nucleus x	Medulla
XII	773	Hypoglossal nucleus	Medulla
y	781	Nucleus y	Medulla
LING	912	Lingula (I)	Cerebellum
CENT	920	Central lobule	Cerebellum
CUL	928	Culmen	Cerebellum
DEC	936	Declive (VI)	Cerebellum
FOTU	944	Folium-tuber vermis (VII)	Cerebellum
PYR	951	Pyramus (VIII)	Cerebellum
UVU	957	Uvula (IX)	Cerebellum
NOD	968	Nodulus (X)	Cerebellum
SIM	1007	Simple lobule	Cerebellum
AN	1017	Ansiform lobule	Cerebellum
PRM	1025	Paramedian lobule	Cerebellum
COPY	1033	Copula pyramidis	Cerebellum
PFL	1041	Paraflocculus	Cerebellum
FL	1049	Flocculus	Cerebellum
FN	989	Fastigial nucleus	Cerebellum
IP	91	Interposed nucleus	Cerebellum
DN	846	Dentate nucleus	Cerebellum
